# Mapping the Vaginal Metabolic Profile in Dysbiosis, Persistent Human Papillomavirus Infection, and Cervical Intraepithelial Neoplasia: A Scoping Review

**DOI:** 10.3390/ph19010042

**Published:** 2025-12-24

**Authors:** Ednéia Peres Machado, Allan Michael Junkert, Raul Edison Luna Lazo, Idonilton da Conceição Fernandes, Fernanda Stumpf Tonin, Luana Mota Ferreira, Helena Hiemisch Lobo Borba, Roberto Pontarolo

**Affiliations:** 1 Postgraduate Programme in Pharmaceutical Sciences, Universidade Federal do Paraná, Jardim Botânico, Curitiba 80210-170, PR, Brazil; edpmach@gmail.com (E.P.M.); allan.junkert@gmail.com (A.M.J.); raulunalazo@gmail.com (R.E.L.L.); idonilton.fernandes@ufpr.br (I.d.C.F.); luanamota@ufpr.br (L.M.F.); helena.borba@ufpr.br (H.H.L.B.); 2 Pharmacy and Pharmaceutical Technology Department, Faculty of Pharmacy, University of Granada, 18012 Granada, Spain; fer_stumpf_tonin@hotmail.com; 3 Department of Pharmacy, Universidade Federal do Paraná, Jardim Botânico, Curitiba 80210-170, PR, Brazil

**Keywords:** metabolome, HPV, dysbiosis, uterine cervical neoplasms, biomarkers

## Abstract

**Background/Objectives:** This scoping review aimed to map evidence on metabolic alterations in the vaginal environment associated with dysbiosis, transient and persistent human papillomavirus (HPV) infection, and cervical dysplasia, highlighting potential metabolic and protein biomarkers for early detection of cervical cancer. **Methods:** Systematic searches were conducted in PubMed, Scopus, and Web of Science, following the JBI methodology and PRISMA-ScR guidelines. Studies jointly evaluating vaginal metabolites and proteins in women with HPV and cervical intraepithelial neoplasia (CIN) in the context of dysbiosis were included. **Results:** After duplicate removal, 196 records were screened, and 41 studies were selected—mostly cross-sectional observational designs—published between 2006 and 2025, predominantly by Chinese research groups. *Lactobacillus* spp. predominated in HPV-negative women, while HPV infection was associated with a dysbiotic environment enriched with anaerobes such as *Gardnerella vaginalis*, *Atopobium vaginae*, *Prevotella*, and *Sneathia*. Of 389 metabolic and protein markers associated with HPV infection and CIN, 44 underwent ROC analysis, with prolineaminopeptidase, 5′-O-methylmelledonal, and calonectin showing high diagnostic performance (AUC > 0.90). **Conclusions:** These results suggest vaginal microbiome and metabolic profiles may represent promising biomarkers for persistent HPV infection. Further, longitudinal studies with larger samples are needed for clinical validation.

## 1. Introduction

Cervical cancer remains one of the most prevalent malignancies among women, with an estimated 661,000 new cases globally in 2022 [1]. Its pathogenesis is predominantly driven by persistent infection with high-risk oncogenic human papillomavirus (HR-HPV), which is detected in up to 95% of cases [2]. Despite advances in preventive strategies, cervical cancer screening continues to face significant challenges. Pap smear cytology exhibits low sensitivity (ranging from 18 to 66%), resulting in high false-negative rates [3]. Although HPV DNA testing is more sensitive and has been recommended by the World Health Organization since 2021 [4], it still yields false-negative results in 5.5% to 11% of cases [5]. These inaccuracies may arise from infections with low viral load, gene deletions during transcription [6], or the use of tests that do not cover all 15 HR-HPV and 11 low-risk HPV (LR-HPV) types [7,8,9]. This scenario is further complicated by the fact that approximately 3% to 8% of adenocarcinomas are truly HPV-negative [7]. In addition, HPV DNA testing cannot distinguish transient from persistent infections and provides no information on cytological lesions, requiring histological confirmation [10]. Moreover, available vaccines, either nonavalent or tetravalent, do not cover all oncogenic HPV serotypes [11], reinforcing the continued need for robust screening programs [12].

In this context, complementary approaches such as metabolomics and metagenomics have emerged as promising tools for improving the detection of HPV infection (both transient and persistent), cervical intraepithelial neoplasia (CIN) grades I–III, and cervical cancer [13,14]. A significant association between vaginal dysbiosis and cervical cancer has been demonstrated [15,16,17,18], with potential microbial biomarkers suggested, such as *Gardnerella* for CIN II [19] and *Sneathia* for persistent HPV [20,21,22,23]. The depletion of *Lactobacillus* spp. and the enrichment of anaerobes including *Gardnerella vaginalis*, *Atopobium vaginae* (*Fannyhessea vaginal*), *Prevotella* spp., *Sneathia* spp., and *Megasphaera* spp. promote the production of bioactive metabolites and bacteriocins that modulate the local immune response in a species-specific manner. This includes altering levels of cytokines pro-inflammatory IL-17, immunoregulatory IL-10, and Th2/anti-inflammatory IL-4, thereby compromising immune efficacy and facilitating HPV persistence [16,24,25] and cervical dysplasia progression [26,27,28,29,30,31,32].

Accordingly, metabolomics has emerged as a promising technique capable of mapping the metabolic profile [33] with high sensitivity in cervicovaginal fluids [34]. It identifies subtle biochemical signatures, such as alterations in sphingolipid, long-chain fatty acid, and amino acid, which reflect the dynamic interaction between pathogens, microbiota, and host [25]. However, critical gaps remain due to the lack of clinically validated metabolic biomarkers able to distinguish transient from persistent HPV infections or different grades of cervical dysplasia [14,33,35,36,37]. These limitations are compounded by methodological variability that undermines reproducibility across studies [36,38,39,40,41] and by the scarcity of multi-omics approaches integrating metabolomics and metagenomics [25,36,42,43].

Given these challenges, this scoping review aimed to map the available evidence on metabolic alterations in the vaginal environment associated with dysbiosis, transient and persistent HPV infection, and progression of CIN, with emphasis on identifying metabolic and protein biomarkers applicable to screening, early diagnosis of high-grade lesions, and risk stratification in HPV-positive women.

## 2. Materials and Methods

This scoping review was conducted in accordance with the JBI Manual for Evidence Synthesis [44] and reported following the Preferred Reporting Items for Systematic Reviews and Meta-Analyses Extension for Scoping Reviews (PRISMA-ScR) guidelines [45]. The study protocol was registered in the Open Science Framework (OSF) and is available at https://osf.io/869xb/ (accessed on 12 December 2025) under the DOI: 10.17605/OSF.IO/869XB.

### 2.1. Research Questions

The research questions were developed based on the PCC acronym. The population considered includes women with transient or persistent HPV infection, pre-neoplastic lesions, or cervical cancer. The concept involves interactions within the vaginal microenvironment, encompassing microbiota and metabolome, with a focus on potential biomarkers for the progression of HPV infection and the development of CIN. The context had no restrictions. From this outline, the following research questions were formulated: (i) Which metabolic profiles and microbial species characterize the vaginal microenvironment during dysbiosis in women with transient or persistent HPV infection, dysplastic lesions, and cervical cancer? (ii) Can vaginal metabolites and proteins serve as biomarkers to distinguish between transient and persistent HPV infection, as well as to identify cervical dysplasia and cervical cancer? and (iii) Are there metabolic signatures and microbiota patterns capable of differentiating HPV-negative CIN from HPV-positive CIN at equivalent stages?

### 2.2. Search Strategy

A comprehensive search was conducted in May 2025 using the PubMed search engine and the Scopus, and Web of Science databases, supplemented by manual screening of the reference lists from included articles and gray literature searches, including theses and dissertations, via Google Scholar. Descriptors related to HPV, cervical intraepithelial neoplasia (CIN), vaginal metabolome, and microbiome were combined using the Boolean operators “AND” and “OR”. The complete search strategies are provided in Appendix A, Table A1.

### 2.3. Inclusion and Exclusion Criteria

The inclusion criteria considered primary studies conducted either in vivo or in vitro, published as scientific articles, theses, or dissertations, that simultaneously evaluated the vaginal microbiota, metabolites, and proteins in the context of HPV infection (transient or persistent) or in association with CIN. To ensure comparability and accuracy of findings, only studies that analyzed microbiota using validated laboratory methods (e.g., microscopy, culture, or 16S rRNA sequencing) and quantified metabolites and proteins using appropriate analytical techniques—whether omics or non-omics approaches—were considered eligible. When a study was available both as a thesis and as a peer-reviewed journal article, the published version was prioritized.

Studies were excluded if they were conference abstracts, articles written in non-Roman characters, studies that did not simultaneously evaluate the vaginal microbiota, the metabolome, and HPV infection/CIN, or investigations focusing exclusively on immune response proteins (such as cytokines, interleukins, chemokines, and antibodies). In addition, because this review focused on data derived from direct laboratory measurement of metabolites and proteins, metagenomic studies that inferred metabolic pathways or gene functions exclusively through bioinformatic analyses—such as ortholog (KO) mapping in databases like KEGG or functional predictions derived from taxonomic profiles (e.g., PICRUSt)—were excluded. Although such approaches are useful for estimating the metabolic potential of the microbiome, they do not provide empirical data on the actual presence or concentration of metabolites in the cervicovaginal environment.

### 2.4. Study Selection

After duplicate removal using EndNote software (version 21, Clarivate Analytics, Philadelphia, PA, USA), records were imported into Rayyan web app, free version, Rayyan Systems, Inc. (Qatar Computing Research Institute) [46], for title and abstract screening. Studies retained after initial screening underwent full text review. Those meeting all inclusion criteria were selected for data extraction. Gray literature documents were identified through manual searches of institutional repositories, academic platforms, and Google Scholar. These records were organized in Microsoft Office Excel 2019 (Microsoft Corporation, Redmond, WA, USA), where they were initially screened by title and abstract, followed by full-text evaluation.

Title and abstract screening, as well as full-text review were conducted independently by two reviewers (EPM and ICF), with disagreements resolved through consensus meetings and, when necessary, by a third reviewer (HHLB).

### 2.5. Data Extraction

Data extraction was performed independently by two reviewers (EPM and AMJ) using a standardized form developed in Microsoft Excel 2019 (Microsoft Corporation, Redmond, WA, USA). The collected information included authors, language, year of publication, country of study, study design, type of sample, laboratory methods employed, all metabolites, proteins, enzymes, and microorganisms evaluated, as well as the main conclusions reported by the authors.

This approach allowed the integration and comparison of results derived from different analytical platforms—including LC-MS, GC-MS, enzymatic assays, spectrometry, proteomics, and 16S rRNA—without requiring artificial data transformations or forced unit standardization. Consequently, the final synthesis was grounded in the biological and functional interpretations provided by the original studies, ensuring consistency across both omics and non-omics approaches.

The data were systematically extracted from the main text, tables, and figures of the included studies, with additional consultation of Supplementary Materials when available. When results were presented in both tables and figures, tabulated data were prioritized due to their greater precision. For information available exclusively in graphical format, numerical extraction was performed using ImageJ software (version 1.44, National Institutes of Health, Bethesda, MD, USA) [47], with the scale bar used as a reference. All measurements were conducted in triplicate to ensure greater accuracy and reproducibility.

### 2.6. Data Synthesis

As this is a mapping study, we did not conduct a formal assessment of methodological quality or risk of bias of the c the visualization of the main trends. Critical interpretation was further developed in the Discussion section, highlighting how the heterogeneity of the studies influenced the robustness of the conclusions.

The results were presented in textual and tabular formats, complemented by various graphical representations. Maps were created to illustrate the geographical distribution, temporal trends of publications, and methodological designs of the studies. Bar charts displayed the frequency of variables according to the total bacterial abundance in relation to HPV status and CIN presence, as well as LDA scores of microorganisms reported in the articles, considering only those identified in two or more studies.

The Sankey diagram synthesized the findings by illustrating the flow between the most frequent microorganisms and the metabolites most strongly associated with this group of bacteria. All analyses were conducted in the R environment, version 4.1.3 [48] using ggplot2, version is 4.0.0 [49].

## 3. Results

**Figure 1:**
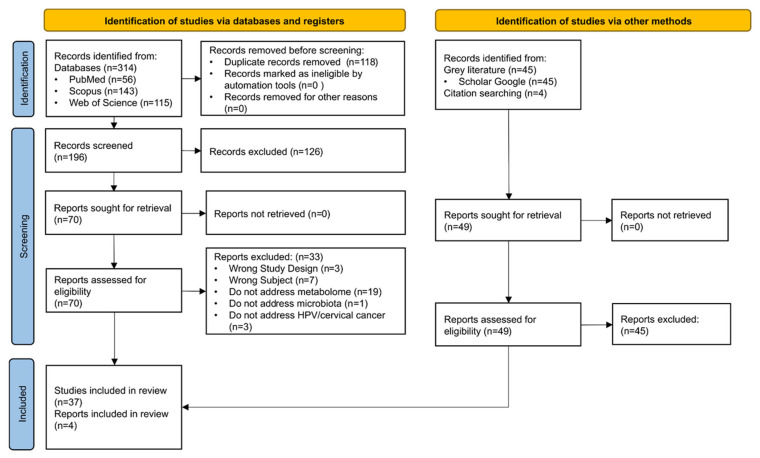
PRISMA flow diagram of the scoping review [50].

Of the 314 studies initially identified, 118 duplicates were removed. Among the 196 screened articles, 126 were excluded due to inconsistency with the inclusion criteria. Of the 70 studies assessed in full text, 37 were eligible, with an additional four articles identified through reference screening, resulting in a total of 41 included studies. No theses or dissertations were included (Figure 1). The list of excluded studies, along with the reasons for exclusion is provided in Supplementary Materials, Table S1. 

### 3.1. Characteristics of the Included Studies

**Figure 2:**
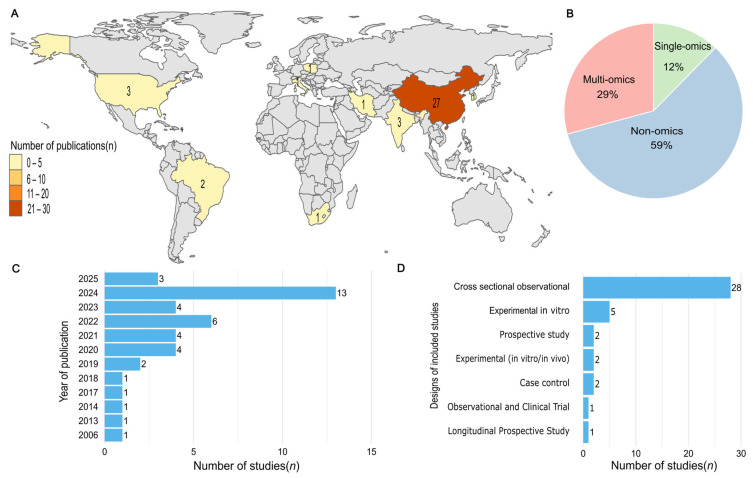
(**A**)—Geographical distribution of the included studies; (**B**)—Classification of studies by analytical approach: multi-omics, single-omics, and non-omics; (**C**)—Distribution of studies by publication year; (**D**)—Methodological design used in the studies.

Most studies were conducted in China (*n* = 27; 65.8%), with the highest concentration of publications in 2024 (*n* = 13; 31.7%). In vivo studies predominated (*n* = 34; 82.9%), particularly cross-sectional observational designs (*n* = 28; 68.3%), while five studies (12.9%) were in vitro and two (4.9%) employed hybrid in vivo/in vitro approaches. Only one study was a multicenter in design (14). Regarding analytical approaches, non-omics techniques were used in 24 studies (58.5%), while 12 (29.3%) employed multi-omics methodologies (Figure 2, Table 1).

Exclusion criteria for potential confounders of the microbiota or metabolic profiles such as recent sexual activity, pregnancy, antibiotic use, and severe immunological disorders—were reported in 21 studies (51.2%). These criteria aimed to eliminate variables that could artificially alter the vaginal environment, thereby hindering the accurate identification of metabolic or microbial changes associated with dysbiosis, HPV infection, and CIN (Appendix A, Table A2).

Cervical scrapings were the predominant sample type for HPV detection (*n* = 30; 73.2%), while vaginal swabs were preferred for microbiota analysis (*n* = 36; 87.8%) and for the assessment of metabolites and proteins (*n* = 21; 51.3%). Cytology and histology were performed in 60.9% (*n* = 25) of the studies (Supplementary Materials, Table S2).

HPV detection was primarily performed using Hybrid Capture 2 (HC2) (*n* = 7; 17.1%), genotyping assays (*n* = 5; 12.2%), reverse dot blot PCR, and Roche Linear Array (*n* = 4; 9.75%). In vitro studies utilized HeLa (HPV18+) [51,52,53], SiHa [51,52,54], and CasKi (HPV16+) [54,55] cell lines obtained from ATCC and BEI-Resources, cultured in DMEM and RPMI media (Supplementary Materials, Table S1). The cervicovaginal microbiota was mainly assessed through smear microscopy (*n* = 20; 48.8%) and 16S rRNA gene sequencing (*n* = 14; 34.1%). In vitro studies analyzed *Lactobacillus* and anaerobic strains from ATCC and BEI-Resources, cultured in MRS, TSA, TSB, and BHI media [51,53,54] (Supplementary Materials, Table S3).

Metabolite and protein analysis were performed using colorimetric enzymatic methods (n = 21; 51.2%) and liquid or gas chromatography coupled with mass spectrometry (LC-MS/GC-MS) (n = 14; 34.1%) (Supplementary Materials, Table S4). Cytology primarily employed Papanicolaou staining (n = 11; 26.8%), while histology used hematoxylin and eosin (n = 12; 29.3%).

A total of 31,494 samples from women aged 14 to 91 years were analyzed by the authors. HPV was assessed in 38 studies (92.7%), of which 25 (63.2%) also evaluated cellular lesions, and 3 studies (7.2%) exclusively analyzed CIN [56,57,58,59]. There were 20,838 HPV-negative and 9299 HPV-positive samples (Table 1). Two studies addressed transient HPV infection [56,60]; however, only one of them [60] clearly defined the criteria for transient and persistent infection based on a subsequent follow-up of ≥6 months. Among the 7582 cytological/histological analyses, there were 1860 were of CIN I (cervical intraepithelial neoplasia grade I), 1918 CIN II/III, 28 carcinomas in situ (CIS), and 325 invasive carcinomas (CC); no glandular lesions were reported. In total, 389 metabolites and proteins showed statistically significant associations with HPV and cellular lesions, along with 1775 microbial correlations identified. The ROC curve demonstrated good discriminatory power for 44 biomarkers [57,59,60,61,62] (Supplementary Materials, Table S5).

### 3.2. Microbial Diversity and Composition

Findings on alpha and beta diversity were inconsistent, owing to some studies reported increased diversity in HPV-positive cases, CIN, and CC groups [25,36,57,63,64,65,66], while others observed no significant differences [38,61,67,68]. Similarly, beta-diversity analyses showed distinct microbial compositions between HPV-positive, lesion, and control group [52,57,62,65,66,69], though some studies did not confirm this distinction [36,61,70]. A subset of studies identified a qualitative reduction in diversity and density of microbiota with increasing lesion severity or HPV presence [4,63,71,72], whereas others reported no significant differences [38,67,73,74,75].

**Figure 3:**
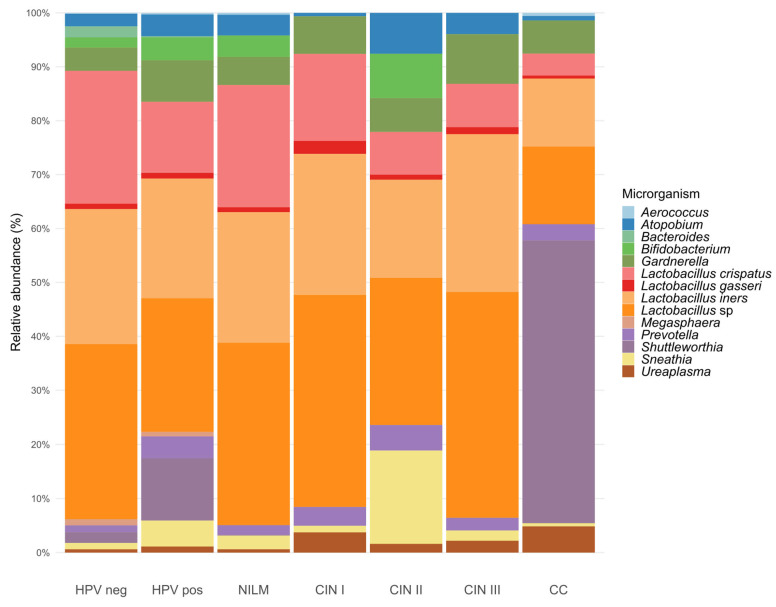
Relative abundance of predominant vaginal microorganisms according to HPV status and CIN grade. The stacked bar chart illustrates the distribution of the most frequently reported bacterial taxa across clinical groups (HPV-negative and HPV-positive (n = 9) [36,52,60,61,62,64,69,73,78], NILM- Negative for Intraepithelial Lesion or Malignancy (n = 3) [52,62,69], CIN I-Cervical Intraepithelial Neoplasia grade I (n = 1) [62], CIN II-Cervical Intraepithelial Neoplasia grade II (n = 2) [62,69], CIN III- Cervical Intraepithelial Neoplasia grade III, and CC-Cervical cancer (n = 1) [62]. The figure illustrates the progressive shift from *Lactobacillus* spp.-dominated communities toward dysbiotic microbiota enriched with anaerobic genera such as *Gardnerella*, *Atopobium*, *Prevotella*, *Sneathia*, *Megasphaera*, and *Shuttleworthia* as lesion severity increases [38,52,61,64,65,69,73,78].

Regarding relative abundance, data extracted from the included studies demonstrated that HPV-negative women exhibited a vaginal microbiota predominantly composed of *Lactobacillus* spp. [14,24,42,52,57,61,62,64,65,66,68,71,72,73,76], particularly *Lactobacillus crispatus* [52,64,73] and *Lactobacillus gasseri* [56], whose frequency decreased with the progression of dysplastic lesions [57,62]. In contrast, *Lactobacillus iners* remained predominant across different groups (HPV-negative, HPV-positive, CIN, and cervical cancer) [62,69,77], reaching up to 50% abundance in CIN II cases. These findings are summarized in Figure 3, which presents stacked bar charts representing the frequency of microbial abundance variables observed in at least two studies, stratified by HPV status (positive/negative).

In the context of transient HPV infection, findings indicate that it already occurs within a microecological imbalance, characterized by a reduction in *Lactobacillus* spp. abundance which, although significant, is less pronounced than that observed in persistent HPV infection and does not entail structural loss of microbial diversity [60].

Among HPV-positive women with CIN, data extracted from the included studies revealed a reduction in *Lactobacillus* spp. and a significant increase in *Gardnerella* spp. [52,60,61,62,64,65,68,78], *Atopobium* spp. (currently *Fannyhessea*) [52,60,62,64,65,66,68,69], *Sneathia sanguinegens* [36,52,60,61,62,64,66,68,69], and *Prevotella bivia* [62,68,69], as well as higher frequencies of *Dialister*, *Megasphaera*, *Shuttleworthia*, *Mycoplasma*, *Ureaplasma*, and *Streptococcus* were reported [36,52,60,61,62,65,66,68,71,76]. In addition, bacterial vaginosis (BV) and aerobic vaginitis (AV) showed significant associations with high-risk HPV (HR-HPV) and with low- and high-grade squamous intraepithelial lesions (LSIL and HSIL) [63,72,78,79]. These findings are summarized in Figure 3, which displays stacked bar charts representing the relative abundance of microorganisms reported in at least two studies, stratified according to lesion status.

**Figure 4:**
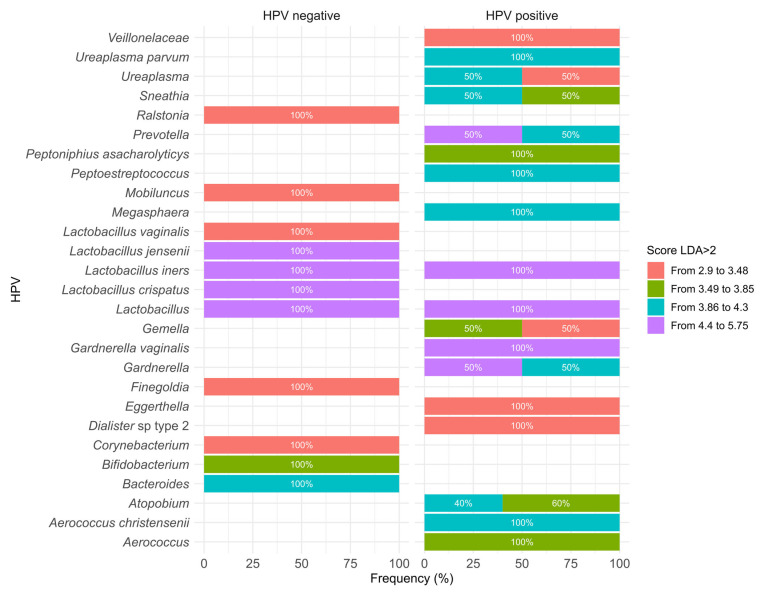
Differential distribution of key vaginal microorganisms according to HPV status, based on LDA scores. The bar chart displays taxa with LDA > 2 reported across the included studies, illustrating their frequency in HPV-negative and HPV-positive groups. Several taxa were reported in more than one study, including: *Lactobacillus* [52,60,61,64] (*n* = 4), *Atopobium* [42,60,61,64] (*n* = 4); *Ureaplasma* [61,68] (*n* = 2), *Sneathia* [60,64] (*n* = 2), *Prevotella* [60,64] (*n* = 2), *Lactobacillus iners* [61,68] (*n* = 2), *Gemella* [42,60] (*n* = 2) *Gardnerella* [61,68] (*n* = 2). Other taxa were reported in a single study, including: *Veillonellaceae*, *Lactobacillus vaginalis*, *Lactobacillus jensenii*, *Lactobacillus crispatus*, *Gardnerella vaginalis*, *Finegoldia*, *Eggerthella*, *Dialister* sp. Type 2, *Aerococcus christensenii* [42], *Ureaplasma parvum*, *Aerococcus* [61], *Ralstonia* [68], *Peptostreptococcus* [52], *Mobiluncus* [60] and *Megasphaera* [25,64]. Overall, the figure demonstrates the predominance *of Lactobacillus* spp. in HPV-negative samples and the enrichment of anaerobic or dysbiosis-associated taxa in HPV-positive samples. Notably, *Lactobacillus iners*, reported in two independent studies, appears in both HPV-negative and HPV-positive contexts, reflecting its well-known ecological ambiguity. Colors indicate LDA score intervals, representing the strength of each taxon’s discriminatory contribution.

The linear discriminant analysis (LDA), performed using the Linear Discriminant Analysis Effect Size (LEfSe) developed by the Huttenhower Lab (Harvard T.H. Chan School of Public Health, Boston, MA, USA) tool, is useful for identifying microbial taxa that act as potential biomarkers with LDA scores > 2.0, reflecting features associated with HPV detection status in the vaginal microenvironment [42,62,66]. Among the included studies (n = 6), LDA identified *Lactobacillus* spp. as being associated with the HPV-negative group [42,52,61,64], particularly *Lactobacillus crispatus* (LDA = 5.75) [42]. In contrast, *Lactobacillus iners* exhibited an ambiguous pattern, being associated with both HPV-negative and HPV-positive groups [60,68]. Among HPV-positive women, significant enrichments were observed for *Gardnerella* [42,61,68], *Sneathia* [62,70], *Atopobium* [42,60,61,64], *Prevotella* [60,64], and *Ureaplasma* (LDA ≥ 4.0) [61,68]. These findings are summarized in Figure 4, which presents bar charts of LDA scores for microorganisms associated with HPV status (positive/negative).

### 3.3. Metabolomics, Functions, and Correlations with Microorganisms

In HPV infection and CIN progression, the most significantly impacted pathways were amino acids, peptides, and analogs (*n* = 92) [25,36,42,57,60,61,66,68,77,85], glycerophosphocholines (*n* = 28) [25,42,57,61,68,70,77], carbohydrates (*n* = 27) [42,57,60,65,66,68,69,70], and fatty acids (*n* = 25) [25,42,57,60,61,69,70,77]. The enzymes sialidase (*n* = 17) [24,38,59,63,64,67,71,72,73,74,76,79,80] and β-glucuronidase (*n* = 9) [24,39,59,72,73,79,81] were enriched in HPV-positive, CIN, and CC groups, while leukocyte esterase exhibited dual behavior (*n* = 12) [24,39,63,72,73,74,75,78,79,80].

In transient HPV infection, studies observed an association with a more stable and protective vaginal environment [56,60], whereas persistence was characterized by greater dysbiosis and increased enzymatic activity, with lower levels of prolyl aminopeptidase (20.56% vs. 54.78% in persistent infection) and N-acetylglucosaminidase (21.57% vs. 53.04% in persistent infection) when compared with persistent HPV infection [56].

Among the metabolites, hydrogen peroxide showed ambiguous behavior (*n* = 11) [24,39,59,62,73,74,75,80,81], being depleted in HPV-positive and CIN groups but enriched in CC [69]. Succinate was enriched in HPV-positive and CIN [36,42] but depleted in HPV-positive samples in other studies [57,61,66]. Lactic acid was decreased in HPV-positive and CIN groups [52,61,77], and increased in CC [58,68]. Maltopentaose [25,42], myo-inositol [61,69], oxidized/reduced glutathione [42,61,78], inosine [42,77], 3-methylxanthine [42,69], 7-methylxanthine [42,69], pipecolate [25,61], catalase [63,78], 3-hydroxyhippurate [25,69], N(1)-acetylspermine [25,69], tryptophan [25,66], and spermidine [25,66] also demonstrated ambiguous behaviors, reflecting complex interactions between the vaginal microbiota and the local inflammatory response [25,36,57,60,61,65,66]. (Supplementary Materials, Table S6).

**Figure 5:**
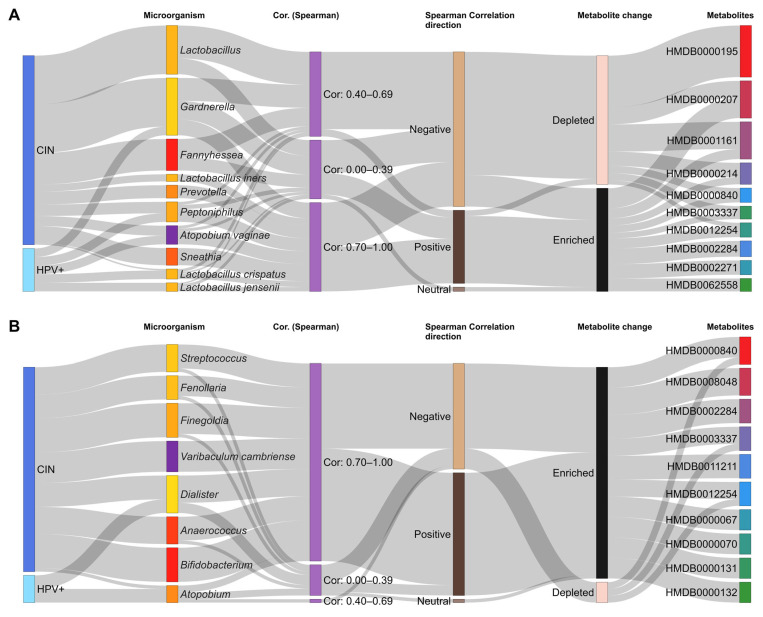
The Sankey diagram depicts the hierarchical relationships among biological categories, with each vertical block representing a node, corresponding to a lesion status, microorganism, Spearman correlation strength, correlation direction, metabolic change, and metabolite identity. The connecting flows (shaded areas) represent the direction and relative weight of the associations, with source nodes indicating the starting element (microorganism or correlation category) and target nodes representing the resulting metabolic outcomes. The width is proportional to the number or weight of the observed associations, enabling visualization of how multiple microorganisms collectively influence metabolic enrichment or depletion rather than acting through isolated linear relationships. Panel (**A**) displays the 10 most frequently reported microbial taxa and their associated metabolites, whereas panel (**B**) presents taxa ranked from 11th to 18th. A selective approach was applied to enhance visualization. LEGEND: CIN—cervical intraepithelial neoplasia; HPV—Human Papillomavirus; Cor—Spearman correlation (weak: 0.00–0.39; moderate: 0.40–0.69; strong: ≥0.70); HMDB—Human Metabolome Database code: HMDB0000195—Inosine, HMDB0000207—Oleic acid, HMDB0000214—DL-Ornithine, HMDB0001161—Deoxycarnitine, HMDB0000840—2-Hydroxyhippurate, HMDB0003337—Glutathione (oxidized), HMDB0012254—Maltopentaose, HMDB0002271—Imidazole propionate, HMDB0002284—N-Acetylcadaverine, HMDB0062558—Pyroglutamine, HMDB0060067—CMP-2-aminoethyl phosphate, HMDB0000070—Pipecolic acid, HMDB0000131—Glycerol, HMDB0000132—Guanine, HMDB0008048—1-Stearoyl-2-arachidonoyl-GPC (18:0/20:4), HMDB0011211—1-Palmitoyl-2-linoleoyl-GPC (16:0/18:2).

The correlation between metabolites and the microbiota demonstrated that certain phospholipids were enriched in the presence of *Lactobacillus* spp. and depleted in dysbiotic microbiota associated with HPV [42,68]. The progression of CIN also appeared to induce linear trends, with metabolites being depleted during initial infection and subsequently enriched in cervical cancer [66]. Alpha-linolenic acid was positively correlated with *Sneathia* spp. and negatively correlated with *Lactobacillus* spp. [61], whereas succinic acid showed a positive correlation with *Klebsiella* and *Sneathia*, and a negative correlation with *Gardnerella* and *Veillonella* [66]. In addition, a microbiota dominated by *Lactobacillus* formed a distinct cluster from non-*Lactobacillus* communities [57]. These findings were synthesized in the Sankey diagram (Figure 5), constructed from the ten most frequent bacterial taxa and, subsequently, the ten metabolites most strongly associated with this group of microorganisms. (Supplementary Materials, Table S7).

However, the extreme complexity and dynamic nature of the cervicovaginal microenvironment—characterized as a system in which microorganisms and host cells (both epithelial and immune) are in constant biochemical interaction—results in the simultaneous production, consumption, and depletion of metabolites [57,63,70]. Thus, the metabolic profile of the cervicovaginal environment is not solely a consequence of the microbiota, but rather the integrated outcome of interactions among HPV, the host, and the vaginal microbiota [70], and these interactions collectively shape cancer risk [57]. Microbial interactions are also highly complex, involving competition for metabolic resources [36], as well as the ability to produce, utilize, and consume individual metabolites [70,73].

The studies evaluated the relationships between bacteria and metabolites using the Spearman correlation coefficient, which capture both the strength and direction (positive or negative) of the association between microbial abundance and metabolite concentrations within the cervicovaginal ecosystem in the presence of HPV infection and CIN [86]. However, some correlations may be influenced by external factors affecting both variables, and large datasets, may reach statistically significant yet weak correlations, necessitating cautious biological interpretation [87]. Of the 1773 correlations identified between bacteria and metabolites, 1057 (59.6%) were assessed using the Spearman correlation coefficient. Among these, 96 (9.1%) were classified as strong, 201 (19.0%) as moderate, and 760 (71.9%) as weak, indicating that most associations exhibited low to intermediate magnitude. As illustrated in the Sankey Diagrams (Figure 5A,B), the resulting metabolic profiles arise from the integrated effects of multiple microorganisms rather than the action of any single taxon, reflecting the dynamic, competitive, and interdependent nature of the cervicovaginal microenvironment.

The patterns observed (Figure 5) further show that the relationships between microorganisms and metabolites within the cervicovaginal ecosystem are neither linear nor unidirectional. For example, although *Prevotella* exhibits a strong negative correlation with certain metabolites during the progression of CIN—an association that would ostensibly suggest depletion—these metabolites appear enriched in the final network output. This apparent paradox underscores the highly interdependent and competitive structure of the cervicovaginal microenvironment.

Moreover, host epithelial and immune cells also contribute to metabolite production and transformation, reinforcing that the final metabolic profile reflects the integrated outcome of microbial interactions, substrate competition, and the host-derived metabolic activity (Figure 5).

### 3.4. In Vitro Studies

In vitro studies [51,53,54,55] and hybrid in vivo/in vitro studies [52,83] demonstrated that *Lactobacillus* strains exerted antiproliferative effects on HeLa, SiHa, and CasKi cells. *Lactobacillus iners* inhibited the proliferation and migration of SiHa cells through activation of the Wnt/lactate-Gpr81 pathway and increased fucosylation [52]. *Lactobacillus crispatus*, *Lactobacillus jensenii*, and *Lactobacillus gasseri* induced cell cycle arrest in CasKi cells [55]. Metabolites from 12 *Lactobacillus* species showed a dose-dependent antiproliferative effect on SiHa and HeLa cells via the production of L-lactic acid and hydrogen peroxide, upregulating E-cadherin expression and reducing MMP9 [51]. However, Qulu et al. [83] observed no differences in MMP concentrations when comparing women with and without HPV infection in the general population, except for elevated MMP10 in HPV-positive women. *Lactobacillus* spp. produced less acetic acid and more butyric and valeric acids compared to vaginal anaerobes [54]. Supernatants of *Lactobacillus gasseri* and *Lactobacillus crispatus* reduced caspase-3 activity in HeLa cells [53]. The expression of indoleamine 2,3-dioxygenase (IDO) was increased in keratinocytes expressing HPV16 E6/E7 oncogenes [82].

### 3.5. Biomarkers

Finally, ROC curves identified proline aminopeptidase and acetylglucosaminidase with high diagnostic accuracy for persistent HPV [59], 5′-O-methylmelledonal and calonectin as discriminators for HPV16 and HPV18 subtypes [60], and oxidized glutathione with an AUC > 0.8 in differentiating normal cervix from HSIL and CC [57] (Supplementary Materials Table S5). A detailed synthesis of microbial and metabolomic findings stratified by clinical group (e.g., HPV-negative, transient HPV infection, persistent HPV infection, NILM, CIN I, CIN II/III, CIS, and CC) is presented in Supplementary Materials Table S8.

## 4. Discussion

The relationship between HPV and the vaginal microbiota is complex and bidirectional, sustained by metabolic alterations that directly influence viral persistence and the local immune response [75]. These findings support the existence of a vicious cycle of dysbiosis–immunosuppression–lesion progression [58], in which the depletion of protective *Lactobacillus* species, combined with the enrichment of anaerobes, leads to reduced levels of lactate and hydrogen peroxide, increased vaginal pH, formation of cooperative biofilms, enhanced sialidase activity, and the accumulation of metabolites such as putrescine and succinate [35,36,41]. These shifts promote an immunosuppressive Th2-skewed microenvironment [64,68], compromising the mucosal barrier and facilitating viral persistence. Furthermore, evidence suggests that HPV itself can modulate epithelial metabolism independently of the microbiota, reinforcing this cycle and contributing to a microenvironment conducive to HPV persistence and cervical carcinogenesis [36,57,67].

The results revealed that HPV-negative women exhibit a predominance of *Lactobacillus* spp. [60], particularly *L. crispatus*, *L. gasseri*, and *L. jensenii*, which are associated with the maintenance of vaginal homeostasis [56,88,89,90]. In contrast, the role of *L. iners* was inconsistent, as it was associated both with high-risk profiles [21,27,31,32,91,92,93] and with antitumor effects in vitro [52], suggesting that its function may vary according to individual host characteristics [36].

Studies have shown that dysbiosis—characterized by the enrichment of anaerobes such as *Gardnerella vaginalis*, *Atopobium*, *Sneathia*, *Megasphaera*, *Prevotella*, *Corynebacterium*, *Peptoniphilus*, *Finegoldia*, and *Streptococcus*, among others [91]—together with a reduction in *Lactobacillus*, is observed in transient HPV infections. The transition to persistent infection appears to be marked by a subsequent increase in *L. iners*, *L. crispatus*, and *Aerococcus* [94]. The formation of polymicrobial biofilms and the ability of these microorganisms to negatively modulate the local immune response are strategies that facilitate viral persistence in the cervical epithelium [14,25,36,42,51,54,58,62,63,65,73,85].

Conversely, dysbiosis was characterized by an enrichment of anaerobes such as *Gardnerella vaginalis*, *Atopobium*, *Sneathia*, *Megasphaera*, *Prevotella*, *Corynebacterium*, *Peptoniphilus*, *Finegoldia*, and *Streptococcus*, among others [94], all significantly associated with persistent HPV infection and progression of CIN. The formation of polymicrobial biofilms and the ability to negatively modulate the immune response are strategies that facilitate viral persistence in the cervical epithelium [14,25,36,42,54,57,61,65,66,68,70,76,82].

The functional differences among *Lactobacillus* species appear to be related to their lactic acid production profiles. *L. crispatus*, *L. gasseri*, and *L. jensenii* predominantly synthesize D-lactate, which increases the viscosity of the cervicovaginal mucus, aiding in the retention of viral particles, whereas *L. iners* produces L-lactate, associated with MMP-8 activation and degradation of the mucosal barrier [88,89,90,95,96,97]. However, an in vitro study reported predominant L-lactate production by *L. crispatus*, *L. gasseri*, and *L. jensenii* [51], in apparent contradiction with other studies [8,66,80]. Additionally, it observed that different *Lactobacillus* species reduced MMP-9 expression in HeLa cells but not in SiHa cells, with *L. vaginalis* showing the strongest antiproliferative effect [51].

Another protective factor is the production of hydrogen peroxide by *L. crispatus*, *L. gasseri*, and *L. jensenii* [31,97,98,99,100], which can induce selective apoptosis of transformed cells through superoxide anions generated by through interaction with myeloperoxidase [31,99,101,102]. However, this role remains controversial [84], since hydrogen peroxide synthesis depends on oxygen availability [31,84], which is reduced under anaerobic conditions such as bacterial vaginosis [31,84]. In such scenario, fermentation and lactic acid production become the predominant mechanisms [103].

Immunomodulatory effects also vary: *L. gasseri* and *L. jensenii* stimulate IFN-γ production, thereby enhancing antiviral response [54], while D-lactic acid downregulates Toll-like receptor (TLR) expression [80,97], increases IL-10, IL-12 [54,104], and IL-23 [51], limits NK cell cytotoxicity [22,105,106], inhibits differentiation of cytotoxic CD8+ T cells, promotes M2 macrophage polarization [104], and stimulates IL-1Ra [107]. In contrast, *L. iners* is associated with an increase in IL-33 levels, which may suppress the adaptive T-cell response [108].

HPV-positive women exhibited low concentrations of glutathione, glycogen, and phospholipid-related metabolites [42]. Oxidized glutathione emerged as a discriminatory marker between normal cervix, CIN III, and cervical carcinomas, with its reduction being associated with increased oxidative stress and potential carcinogenesis [42]. Other lipids, such as glycerophosphorylcholine, 3-hydroxydecanoate, and choline phosphate were found to be reduced in HPV-positive women [42], while 3-hydroxybutyrate showed a strong correlation with *Streptococcus*, *Prevotella*, and *Atopobium vaginae* [25]. Conversely, Lyso PA 12:0 was increased in HPV-positive women without vaginitis [68].

Metabolites such as acetate, proline, and threonine were predicted as microbial products, whose synthesis depends on the enzymes acylphosphatase, prolyl aminopeptidase, and threonyl-tRNA synthetase, which are implicated in neoplastic cell proliferation [36]. Meanwhile, the increase in organic acids and enzymes damages the vaginal epithelium, degrades cervical mucus, and cleaves IgA [76]. In addition, metabolites associated with oxidative stress, such as valyl-glutamate, N,N′-diacetylbenzidine, and oxidized glutathione itself, were identified as discriminators among different grades of cervical dysplasia [57]. In invasive cervical cancer samples, high levels of nucleotides (3′-UMP, cyclic pyranopterin monophosphate, guanosine monophosphate, and 2′-deoxyguanosine 5′-monophosphate) indicated intense DNA degradation due to cellular proliferation and necrosis [57].

From an immunological perspective, bacterial vaginosis was associated with increased IL-1β and reduced IL-17 [63,64], favoring HPV infection and lesion development [63]. In patients with CIN, there was a progressive reduction in IL-2, an increase in IL-10, a decrease in IgA, and an increase in IgG in HPV-positive individuals [71]. Enzymes such as mucinase, sialidase, and proteases were elevated in HR-HPV and dysplasia [76], with sialidase being associated with *Gardnerella* [38,75]. Prolyl aminopeptidase and acetylglucosaminidase, in addition to participating in amino acid metabolism and cell division, were associated with vaginal inflammatory diseases and may favor persistent HPV infection [59].

Studies have also reported that indoleamine 2,3-dioxygenase (IDO) and tryptophan dioxygenase (TDO) act as immunosuppressive mechanisms in HPV-infected epithelium, where leukocytes, particularly PMNs, are important sources of these enzymes in CIN and cancer [82]. Phospholipids and sphingolipids were correlated with local inflammation, even in the absence of cancer [82], while bioactive sphingolipid-derived metabolites may activate TNF-α, intensifying inflammatory signaling [82]. Macrophage migration inhibitory factor (MIF) was identified as a predictive marker of vaginal pH and dysbiosis, as well as a potential contributor to cervical carcinogenesis, along with IL-6, IL-10, and MIP-1α, which were associated with genital inflammatory scores [82].

Biogenic amines, such as putrescine, ethanolamine, N-acetylcadaverine, and N-acetylputrescine, which are increased in dysbiosis and associated with HPV [42], modulate the immune response by stimulating Th2 cells and the production of IL-10 and TGF-β, thereby creating a favorable environment for HPV persistence and CIN progression [108]. Dysbiotic bacteria may increase IL-17 production, impairing the antiviral Th1 response and favoring Th17 differentiation [54].

Among organic acids, succinate regulates inflammatory pathways by activating the HIF-1α signaling pathway [36,42], while acetate functions as a pro-inflammatory metabolic modulator, promoting immune cell survival and proliferation [36,109,110,111] 113.

Nucleotide alterations include reduced levels of adenine, guanine, and cytosine, along with increases in uracil and pseudouridine, which impair DNA repair and promote mutations, creating a permissive environment for viral persistence and carcinogenesis [110,111]. Lipids also impact the immune system, with alterations in lyso- and monoacylglycerolipids and in eicosenoic acid being associated with increased levels of IL-1α, IL-1β, IL-6, and TNF-α [42,111]. These changes contribute to chronic inflammation, creating a permissive microenvironment for HPV immune evasion, thereby favoring its persistence and the progression of CIN [22,32].

This study identified 389 metabolites/proteins with statistically significant associations with HPV infection and CIN, of which 44 demonstrated discriminative capacity via ROC curves analysis, demonstrating high diagnostic performance (AUC > 0.8) with emphasis on proline aminopeptidase, 5-O-methylmelledonal, and calonectrin (AUC > 0.9). However, this evidence highlights a point of tension among authors: while some studies emphasize that the metabolome primarily reflects the microbial community rather than HPV itself [42,57], others propose that the metabolome is a more sensitive and robust biomarker for characterizing environmental and functional changes in the vaginal microenvironment associated with HPV infection and CIN development [25,68,77].

Current research suggests that alterations in the microbiota and metabolic profiles may serve as useful biomarkers, paving the way for novel diagnostic and therapeutic strategies. Given the metabolic complexity of the cervical environment, a combined biomarker panel is recommended over the use of a single marker [37].

The identification of metabolites and proteins with high diagnostic accuracy reinforces the potential of vaginal metabolomics and proteomics to enhance cervical cancer screening protocols, particularly in risk stratification among HPV-positive women. A metabolite panel could serve as a secondary triage tool, identifying women with a higher likelihood of persistent infection or progression to high-grade lesions [112,113]. However, important challenges remain, including the high cost and technical complexity of metabolomic approaches, the need for standardized procedures for sample collection, transport, and storage, and the assurance of reproducibility across different populations and analytical platforms. To enable clinical implementation, longitudinal and multicenter studies are essential to validate diagnostic cut-offs and confirm the predictive value of these biomarkers, in addition to cost-effectiveness analyses [113,114] and the development of simplified assays—such as ELISA-based kits or biosensors—that would allow their large-scale implementation.

The findings of this scoping review should be interpreted with caution due to substantial methodological heterogeneity, variation in analytical platforms, and inconsistencies in clinical characterization, which limit comparability and weaken biological and clinical inferences. Thus, the observed patterns should be viewed as exploratory rather than causal, underscoring the need for more standardized and longitudinal studies.

The limitations of the present review include studies conducted in single-center settings with non-representative samples. Incomplete clinical information (e.g., HPV viral types, hormonal data, distinction between transient and persistent infection) hindered more detailed comparisons. The predominantly cross-sectional nature of the included studies prevented the establishment of cause–effect relationships between alterations in the microbiota, metabolome, and infection progression, as well as the evaluation of transient HPV infection. The use of 16S rRNA sequencing provides low species-level resolution, limiting taxonomic characterization [115]. Some studies assessed only specific enzymes or bacterial groups, often relying on low-resolution analytical approaches. Uncontrolled confounding factors such as diet, menstrual cycle, and menopause may have influenced the results, particularly in studies evaluating serum and urine metabolomic profiles. In addition, in vitro studies have important limitations, as they do not fully reproduce the complexity of the cervicovaginal environment. Despite these limitations, the integration of microbiome and metabolome data emerges as a promising approach for understanding the mechanisms involved in viral persistence and cervicovaginal alterations.

Another limitation identified in this review is the lack of systematic control for key confounding factors that profoundly modulate the vaginal microenvironment. As evidenced by our results, fewer than half of the included studies controlled for variables such as recent sexual activity, antibiotic use, and hormonal status, which can introduce substantial bias into the interpretation of metabolomic and microbiota findings. Sexual activity—particularly exposure to semen—transiently increases vaginal pH, introduces exogenous microorganisms, and adds a significant load of seminal metabolites (e.g., spermine, putrescine), which may mimic or obscure metabolic signatures associated with dysbiosis or HPV infection [116]. Recent antibiotic use is also an important driver of iatrogenic dysbiosis, as it reduces protective *Lactobacillus* species and may generate a microbial profile resembling bacterial vaginosis [117]. Hormonal fluctuations throughout the menstrual cycle, pregnancy, and menopause exert a decisive influence on the vaginal ecosystem: higher estrogen levels promote *Lactobacillus* dominance through increased glycogen deposition and acidic pH, whereas periods of low hormonal activity (menstruation and menopause) are associated with elevated pH and greater microbial diversity [118,119]. Additional factors such as ethnicity, diet, and stress also act as relevant sources of confounding and should be taken into account [120].

Furthermore, the interpretation of these cross-sectional findings is compounded by significant methodological heterogeneity observed across the studies. Second, we observed significant methodological heterogeneity among the included studies, particularly between the large cohort of studies conducted in China and those from other countries. This variability introduces potential biases that affect both the comparability and generalizability of the results. In histopathological analysis, differences in diagnostic criteria and inter-observer variability among pathologists from different healthcare systems may lead to inconsistent classification of cervical lesion grades. Similarly, in metabolomic profiling, studies employed diverse analytical platforms (e.g., different LC-MS or GC-MS systems), sample preparation methods, and metabolite databases. This technical diversity may substantially influence which metabolites are detected and quantified, thereby hindering direct cross-study comparisons. Furthermore, the predominance of Chinese studies in our review, while reflecting current research trends, may limit the extrapolation of findings to other ethnic and geographical populations.

Additionally, it is important to emphasize that the validation of candidate biomarkers requires study designs capable of establishing temporality and reproducibility. Longitudinal studies are essential to determine whether microbial and metabolic alterations precede or result from viral persistence, thereby allowing the assessment of the true predictive value of these biomarkers. Likewise, multicenter studies enhance external validity by testing these markers across different populations, clinical settings, and analytical platforms, an indispensable requirement for their future incorporation into screening, triage, and early diagnostic strategies. Thus, the limitations of the current studies design not only restrict causal inference but also hinder the clinical consolidation of the identified biomarkers.

We highlight that these design limitations do not undermine the promising exploratory patterns identified; rather, they transform them into a clear and urgent research agenda. Our review serves as a starting point, synthesizing the existing evidence to justify and guide investments in robust longitudinal cohorts and multicenter collaborations, which are fundamental for the consolidation and clinical translation of these biomarkers.

## 5. Conclusions

HPV infection and CIN progression are associated with a dysbiotic pattern characterized by a reduction in *Lactobacillus* spp., particularly *Lactobacillus crispatus*, and an increase in anaerobes, notably *Gardnerella vaginalis*, *Fannyhessea vaginae*, *Sneathia*, and *Prevotella.* A total of 389 metabolites and proteins were associated with HPV infection and CIN, with the most significantly impacted pathways involving amino acids, glycerophospholipids, carbohydrates, and fatty acids. Early-stage depletion of hydrogen peroxide, glutathione, and lactic acid, and maltopentaose was observed, with enrichment of succinate, putrescine, N-acetylcadaverine, and other biogenic amines in more advanced disease states.

In this scoping review, 44 metabolites and proteins with high diagnostic performance were identified. Notable biomarkers included proline aminopeptidase and acetylglucosaminidase for persistent HPV infection, 5′-O-methylmelledonal for HPV16, calonectrin for HPV18, and oxidized glutathione for cervical cancer.

Thus, metabolites demonstrated distinct signatures for HPV-positive and HPV-negative cases, as well as across different grades of CIN, suggesting the potential of the metabolome to differentiate the stages of CIN development. However, methodological heterogeneity, the absence of a clear distinction between transient and persistent infection, and the predominance of cross-sectional designs, which prevent the establishment of cause–effect relationships, limit the robustness of clinical inferences.

Despite the limitations, the findings reinforce that the combined profile of microbiota and metabolites in the vaginal environment represents a promising approach for early diagnosis and risk stratification. In this context, an integrated multi-omics approach, particularly in longitudinal and multicenter studies, is essential for validating biomarker panels and their future incorporation into clinical practice.

## Figures and Tables

**Table 1:** Summary of the characteristics of the studies included in the scoping review.

Author, Year	Country	Study Design	Omics Approach	Sample Size	HPV (−), (n)	HPV (+), (n)	Cytology/ Histology Performed	Bacterial Taxa or Strains Associated with HPV-Positive Contexts	Significant Metabolites (HPV/Lesions)
Faktor et al., 2024 [14]	Scotland, Poland and Czech Republic	Cross sectional	Omics	6	0	1	YES	↑ *Lactobacillus iners*, *L. crispatus*, *Prevotella*, *Gardnerella*, *Sneathia*, *Fusobacterium*, *Helicobacter*	↓ Glucose-6-phosphate isomerase, ↓ Pyruvate kinase (HPV+)
Zheng et al., 2019 [24]	China	Cross sectional	Non- omics	532	154	378	YES	↑ BV, *Trichomonas vaginalis*, *Chlamydia* spp. ↓ *Lactobacillus* spp.	↓ H_2_O_2_, ↑ Sialidase, ↑ GUS, ↑ GADP (HPV+/Lesions)
Bokulich et al., 2022 [25]	USA	Cross sectional	Multi- omics	72	18	54	YES	↑ *Prevotella bivia*, *Peptoniphilus*, *Streptococcus anginosus*, *Atopobium vaginae*, *Sneathia sanguinegens*, *Veillonellales*, *Finegoldia*, *Mobiluncus* ↓ *Lactobacillus crispatus*, *L. iners*	↑ 3-Hydroxybutyrate, ↑ Deoxycarnitine, ↑ Pipecolate (HPV+/ICC); ↓ Maltopentaose
Chorna et al., 2020 [36]	Puerto Rico	Cross sectional	Multi- omics	19	8	11	NR	↑ *Lactobacillus* sp., *Atopobium vaginae*, *Gardnerella*, Shuttleworthia ↓ *Lactobacillus iners*, *Megasphaera*	↑ Acetate, ↑ Proline, ↑ Threonine (HPV+); ↑ Succinate (HPV−)
Cheng et al., 2024 [38]	CSO ^b^	Cross sectional	Non- omics	5.099	4.463	636	NR	↑ BV, clue cells↓ *Lactobacillus* (in the normal microbiota)	↑ Sialidase (HPV+)
Liu et al., 2024 [39]	China	Prospective study	Non- omics	466	326	140	YES	↑ BV-associated profile, ↓ *Lactobacillus* spp. (inferred)	↑ GUS, ↑ LE, ↑ SNA (significant only in HR-HPV+ women with CIN1)
Borgogna et al., 2020 [42]	USA	Cross sectional	Multi- omics	39	13	26	NR	↑ *Gardnerella vaginalis*, *Eggerthella*, *Atopobium* (≠A. vaginae), *Dialister* spp., *Gemella* ↓ *Bifidobacteriaceae*, *Atopobium vaginae*	↑ Putrescine, ↑ Ethanolamine, ↓ GSH, ↓ Choline phosphate, ↑ N-acetyl-cadaverine (HPV+)
Pawar et al., 2022 [51]	India	Experimental in vitro	Non- omics	NA	NA	NA	NA	*Lactobacillus vaginalis* and *L. salivarius*—lowest IC50 **** on HPV16+ (SiHa) and HPV18+ (HeLa) cell lines	↑ L-lactic acid, ↑ H_2_O_2_, ↑ E-cadherin, ↓ MMP9 (HPV+ CC)
Fan et al., 2021 [52]	China	Experimental in vitro/in vivo	Multi- omics	119	9	110	YES	Protective: *Lactobacillus iners*—↓ proliferation and migration of cervical cancer cells	↓ Lactate (in vivo); ↑ Core fucosylation (Lactate-induced in vitro, HPV+, cervical cancer)
Motevaseli et al., 2013 [53]	Iran	Experimental in vitro	Non- omics	NA	NA	NA	NA	Protective: *Lactobacillus crispatus*, *L. gasseri*—↓ proliferation of cervical cancer cells, ↓ apoptosis	↓ Caspase-3 activity (in HeLa, HPV18+)
Nicolò et al., 2021 [54]	Italy	Experimental in vitro	Non- omics	NA	NA	NA	NA	Protective: *L. gasseri*, *L. jensenii*, *L. crispatus* Detrimental: *G. vaginalis*, *A. vaginae*, *P. bivia*, *M. micronuciformis*	↑ Butyrate e valerate, ↓ Acetate
Wang et al., 2018 [55]	China	Experimental in vitro	Non-omics	NA	NA	NA	NA	Protective: *L. crispatus*, *L. jensenii*, *L. gasseri*—↓ proliferation of cervical cancer cells,	↓ HPV16 E6/E7, ↓ CDK2, ↓ Cyclin A, ↑ p21, ↓ cell viability (HPV16+)
Kamble et al., 2022 [56]	India	Experimental in vitro	Omics	53	NR	NR	YES	Protective: *Lactobacillus gasseri*, *L. fermentum*, *L. delbrueckii*, *Enterococcus faecium*—antimicrobial activity against pathogens isolated from LSIL, HSIL, and ICC patients	None (no significant association with HPV or lesions)
Ou et al., 2024 [57]	China	Cross sectional	Multi- omics	100	NR	NR	YES	↑ *Gardnerella*, *Prevotella*, *Streptococcus*, *Atopobium* ↓ *Lactobacillus crispatus*	↑ N,N′-Diacetylbenzidine, ↑ Oxidized glutathione (CC); ↑ Valyl-glutamate (CC); ↑ 4-Hydroxydebrisoquine (^l^CIN3)
de Magalhães et al., 2021 [58]	Brazil	Cross sectional	Non- omics	78	78	NR	YES	↑ *Lactobacillus* iners	↑ L-lactic acid (HSIL)
Li et al., 2024 [59]	China	Cross sectional	Non- omics	1.281	772	509	NR	↑ BV ↓ *Lactobacillus* spp.	↑ Prolyl aminopeptidase (HPV+) ↑ Acetylglucosaminidase (HPV+)
Yang et al., 2024 [60]	China	Cross sectional	Multi- omics	26	9	17	NR	↑ *Prevotella*, *Sneathia*, *Atopobium*, *Bifidobacteriaceae* ↓ *Lactobacillus*	↑ 5′-O-Methylmelledonal, ↑ DG(9D3/11M3/0:0), ↑ Glutaminylglutamine, ↑ 2,4-Diisopropyl-3-methylphenol (HPV16+); ↑ Calonectrin, ↑ Longifolonine, ↑ N-Benzylphthalimide (HPV18+)
Zhang et al., 2024 [61]	China	Cross sectional	Multi- omics	42	17	25	NR	↑ *Sneathia amnii*, *Gardnerella*, *Atopobium*, Mycoplasma, Ureaplasma parvum, *Veillonella montpellierensis*, *Aerococcus christensenii* ↓ *Lactobacillus*, *Lactobacillus iners*	↑ 9,10-DiHOME, ↑ α-linolenic acid, ↑ ethylparaben, ↑ glycocholic acid, ↑ prostaglandin F3α, ↑ pipecolic acid, ↓ S-lactoylglutathione, ↓ 3-methylcrotonylglycine (HPV+)
Cheng et al., 2024 [62]	China	Cross sectional	Omics	254	58	196	YES	↑ *Burkholderiaceae*, *Acinetobacter*, *Streptococcus*, *Dialister*, *Anaerococcus* ↓ *Lactobacillus crispatus*, Pelomonas, Ochrobactrum	↓ H_2_O_2_ (HPV+, CIN, CC)
Li et al. 2024 [63]	China	Longitudinal Prospective	Non- omics	1.281	898	383	NR	↑ *G. vaginalis*, *yeasts* (*Candida* spp.); ↓ *L. acidophilus*	↑ GUS; ↑ SNA, ↑ LE (HPV+)
Lin et al., 2022 [64]	China	Cross sectional	Omics	448	310	138	YES	↑ *Gardnerella*, *Prevotella* ↓ *Lactobacillus* spp.	↑ Sialidase (HPV+, CIN+)
Xu et al., 2022 [65]	China	Cross sectional	Omics	40	10	30	YES	↑ *Prevotella*, *Gardnerella*, *Aquabacterium* ↓ *Lactobacillus* spp.	↑ Lipids (CC); ↓ Lipids (LSIL, HSIL)
Shen et al., 2025 [66]	China	Cross sectional	Multi- omics	156	0	156	YES	↑ *Atopobium*, *Sneathia*, Pseudomonas ↓ Streptococcus	↑ N-methylalanine, ↑ phenylacetaldehyde, ↑ glucose-6-phosphate, ↓ Sucrose, ↑ DL-p-hydroxylphenyllactic acid, ↑ guanine (HSIL/HPV+)
Feng et al., 2023 [67]	China	Cross sectional	Non-omics	2.358	1.880	478	NR	↑ BV, *Trichomonas vaginalis* ↓ *Candida* spp. (VVC)	↑ SNA (HPV other subtypes)
Zhao et al., 2025 [68]	China	Case–control	Multi-omics	164	123	41	NR	↑ *Lactobacillus iners*, Mycoplasma (HPV+ with vaginitis) ↑ *Gardnerella*, *Mycoplasma*, *Ureaplasma* (HPV+ without vaginitis)	↑ methionyl-alanine, ↓ lipids (HPV+)
Jimenez et al., 2024 [69]	USA	Cross sectional	Multi- omics	99	20	31	YES	Protective: *Lactobacillus gasseri*, *L. jensenii—*↓ migration and proliferation, ↑ apoptosis	↑ 2-HG, ↑ 4-hidroxibutirato, ↓ Isobutyrilcarnitine, ↓ Histidine (HPV+/Lesions)
Daí et al., 2024 [70]	China	Prospective study	Multi- omics	65	0	65	YES	↑ *Prevotella*, *Streptococcus* ↓ *Lactobacillus* spp.	↑ Glycerophospholipids (MetaG10); ↓ Amino acids and peptides (MetaG1, MetaG5) after HPV+ CIN treatment
Zheng et al., 2020 [71]	China	Cross sectional	Non- omics	532	154	378	YES	↑ BV, VVC, TV; ↓ *Lactobacillus* spp.	↑ Sialidase, ↑ GUS (HPV+)
Wang et al., 2017 [72]	China	Cross sectional	Non- omics	531	176	363	YES	↑ BV-associated microecology; ↓ *Lactobacillus* spp.	↓ H_2_O_2_, ↑ SNA, ↑ LE, ↑ GUS, ↑ GADP (associated with CIN and persistent HPV infection)
Zhang et al., 2022 [73]	China	Cross sectional	Non- omics	420	140	280	NR	↑ BV, VVC, TV; ↓ *Lactobacillus* spp.	↓ H_2_O_2_, ↑ Sialidase, ↑ LE (HPV+)
Tao et al., 2023 [74]	China	Cross sectional	Non-omics	169	0	169	YES	↑ BV-associated profile; ↓ *Lactobacillus* spp.	↑ LE, ↓ SNA, ↓ H_2_O_2_, (HPV+ improvement after focused ultrasound)
Shi et al., 2024 [75]	China	Cross sectional	Non- omics	11.540	8.043	3	YES	↑ BV-associated microecology ↓ *Lactobacillus* spp.	↓ H_2_O_2_, ↑ LE (CIN)
Dasari et al., 2014 [76]	India	Case control	Non- omics	109	NR	19	YES	↑ BV, *Trichomonas vaginalis*, *Candida* spp.; ↓ *Lactobacillus* spp.	↑ Mucinase, ↑ Sialidase, ↑ Protease (CIN III/NCIS/ICC)
Dai et al., 2025 [77]	China	Cross sectional	Multi-omics	43	0	43	YES	↑ *Gardnerella*, *Atopobium*, *Sneathia*, *Dialister* ↓ *Lactobacillus crispatus*	↑ Capric acid, ↑Oleic acid, ↑ Inosine, ↑ DL.3 (4-hydroxyphenyl)lactic acid; ↓ Lactate (LSIL/HSIL/HPV+)
Sun et al., 2023 [78]	China	Cross sectional	Non- omics	512	300	300	YES	↑ *G. vaginalis*, *yeasts* (*Candida* spp.); ↓ *L. acidophilus*	↑ Proline aminopeptidase, ↑ LE, ↑ Catalase (HPV+)
Liu et al., 2024 [79]	China	Cross sectional	Non- omics	2.000	1.759	241	NR	↑ BV, AV (aerobic vaginitis); ↓ *Lactobacillus* spp.	↑GUS; ↑ SNA, ↑ LE (HPV+)
Li et al., 2020 [80]	China	Cross sectional	Non- omics	1.019	839	180	YES	↑ BV-associated profile ↓ *Lactobacillus* spp.	↓ H_2_O_2_, ↑ GUS, ↑ SNA, ↑ LE (HPV16+/CIN)
Lu et al., 2021 [81]	China	Cross sectional and clinical trial	Non-omics	276	120	156	YES	↑ BV-associated profile); ↓ *Lactobacillus* spp. (inferred)	↓ H_2_O_2_, ↑ GUS, ↑ acetilglucosaminidase, ↑ SNA (CIN/HPV+)
Venancio et al., 2019 [82]	Brazil	Experimental in vitro/vivo	Non-omics	165	96	69	YES	Gram-stained bacterioscopy indicates presence of BV, *Candida* spp., and *Actinomyces* in HPV+ samples	↑ IDO (squamous cells, leukocytes—HPV+ HSIL/CC); ↑ TDO (stromal leukocytes—HPV16+)
Qulu et al., 2023 [83]	South Africa.	Cross sectional	Non-omics	243	160	83	NR	↑ BV-associated profile; ↓ *Lactobacillus* spp. (inferred)	↑ MMP-10 (CIN)
Choi et al., 2006 [84]	Korea	Cross sectional	Non- omics	1.138	54	96	YES	No significant association between *Lactobacillus* spp. and HPV+	None (no significant association with HPV or lesions)
Total/Summary	NA	NA	NA	31,494	20,383	9299	7582 (CIN I: 1860; CINII/III:1918; CIS:28; CC:325	NA	NA

Note: USA: United States of America, NR: not reported, NA: not applicable. AV: aerobic vaginitis; BV: bacterial vaginosis; CIS: carcinoma in situ; CC: cervical cancer; CIN: cervical intraepithelial neoplasia; DG: diacylglycerol; GADP: glyceraldehyde-3-phosphate dehydrogenase; GSH: glutathione; GUS: β-glucuronidase; HSIL: high-grade squamous intraepithelial lesion; ICC: invasive cervical cancer; IC50: half maximal inhibitory concentration; IDO: indoleamine 2,3-dioxygenase; LE: leukocyte esterase; LSIL: low-grade squamous intraepithelial lesion; MMP: matrix metalloproteinase; NCIS: non-cervical intraepithelial squamous; SNA: sialidase; TDO: tryptophan 2,3-dioxygenase; TV: Trichomonas vaginalis; VVC: vulvovaginal candidiasis. ↑: Increase in microorganism abundance/Metabolite enrichment. ↓: Decrease in microorganism abundance/Metabolite depletion. The background color was added to enhance readability and avoid confusion when interpreting the data.

## Data Availability

No new data were created or analyzed in this study.

## Supplementary Materials

The following supporting information can be downloaded at: https://www.mdpi.com/article/10.3390/ph19010042/s1, Table S1: Reasons for exclusion after full-text screening. Table S2: Summary of HPV detection methods, genotypes, and sample distribution in included studies. Table S3: Summary of vaginal microbiota analysis and HPV-related bacterial findings. Table S4: Summary of significant metabolites in HPV and cervical intraepithelial neoplasia. Table S5: AUC, Sensitivity, and Specificity Values for Metabolites and Proteins Discriminating HPV and Cervical Intraepithelial Neoplasia. Table S6: Statistically significant metabolomic and proteomic markers in HPV-related cervical intraepithelial neoplasia. Table S7: Correlations Between Metabolites, Vaginal Microorganisms, HPV Status, and cervical intraepithelial neoplasia. Table S8: Summary of Microbiota and Metabolomic Findings by Clinical Group.

## Appendix A

**Table A1:** Search strategy and study retrieval steps for the scoping review (Latest update May 2025).

Database	Query	Search Strategy	Records Identified in Databases	#1 AND #2 AND #3
PubMed	#1	((Dysbiosis [MH] OR dysbios * [TIAB] OR disbios * [TIAB] OR Dys-symbios * [TIAB] OR “Dys symbiosis” [TIAB] OR Dysbacterios * [TIAB] OR Disbacterios * [TIAB] OR Microbiota [MH] OR microbiota * [TIAB] OR Microbial [TIAB] OR Microbiome * [TIAB] OR *Lactobacillus* [MH] OR Lactobac * [TIAB]) AND vagin * [TIAB]) OR “Vaginal Diseases” [MH]	36,041	56
	#2	Alphapapillomavirus [MH] OR Alphapapillomavirus * [TIAB] OR HPV [TIAB] OR “Human Papillomavirus *” [TIAB] OR “Papillomavirus E7 Proteins” [MH]	69,515	
	#3	“Metabolism” [MH] OR Metabolom * [TIAB] OR Metabolit * [TIAB] OR Metabolic [STIAB] OR “lactic acid” [MH] OR “Hydrogen peroxide” [TIAB] OR “Hydrogen peroxid *” [TIAB] OR metalloprotein * [TIAB] OR sialidase [TIAB] OR “reactive oxygen species” [TIAB] OR “Aromatic amines” OR putrescine [TW] OR cadaverine [TW] OR propionate [TIAB] OR triacylglycerol [TIAB] OR acetoacetate [TIAB] OR Trimethylamine * [TIAB]	3,796,575	
SCOPUS	#1	TITLE-ABS-KEY (((dysbios * OR disbios * OR Dys-symbios * OR “Dys symbiosis” OR Dysbacterios * OR Disbacterios * OR microbiota * OR Microbial OR Microbiome * OR Lactobac *) AND vagin *) OR “Vaginal Diseases”)	17,035	143
	#2	TITLE-ABS-KEY (Alphapapillomavirus * OR HPV OR “Human Papillomavirus *” OR “Papillomavirus E7 Proteins”)	82,849	
	#3	TITLE-ABS-KEY (“Metabolism” OR Metabolom * OR Metabolit * OR Metabolic OR “lactic acid” OR “Hydrogen peroxid *” OR metalloprotein * OR sialidase OR “reactive oxygen species” OR “Aromatic amines” OR putrescine OR cadaverine OR propionate OR triacylglycerol OR acetoacetate OR Trimethylamine * OR proteoma OR protein OR enzyme OR bacteriocin)	6,142,866	
Web of Science	#1	(dysbios * OR disbios * OR Dys-symbios * OR “Dys symbiosis” OR Dysbacterios * OR Disbacterios * OR microbiota * OR Microbial OR Microbiome * OR Lactobac *) AND vagin *) OR (“Vaginal Diseases”)	9546	115
	#2	(Alphapapillomavirus * OR HPV OR “Human Papillomavirus*” OR “Papillomavirus E7 Proteins”)	91,229	
	#3	(“Metabolism” OR Metabolom * OR Metabolit * OR Metabolic OR “lactic acid” OR “Hydrogen peroxid *” OR metalloprotein * OR sialidase OR “reactive oxygen species” OR “Aromatic amines” OR putrescine OR cadaverine OR propionate OR triacylglycerol OR acetoacetate OR Trimethylamine * OR proteoma OR protein OR enzyme OR bacteriocin)	3,362,413	

Legend: The asterisk (*) was used as a truncation symbol in the search strategy to capture variations of the same root word and maximize search sensitivity. The background color was added to enhance readability and avoid confusion when interpreting the data.

**Table A2:** Sample exclusion criteria adopted by the studies in vivo included in the scoping review.

Exclusion Criteria	Number of Studies	References
Pregnant women	15	Borgogna et al., 2020 [42], Li et al., 2024 [59], Liu et al., 2024 [79], Zhang et al., 2024 [61], Li et al., 2024 [63], Lin et al., 2022 [64], Cheng et al., 2024 [62], De Magalhães et al., 2022 [58], Xu et al., 2022 [65], Bokulich et al., 2022 [25], Shi et al., 2024 [75], Feng et al., 2023 [67], Qulu et al., 2023 [83], Dai et al., 2025 [77], Zhao et al., 2025 [68].
Lactating women	4	Borgogna et al., 2020 [42], Li et al., 2024 [63], Zhang et al., 2024 [61], Kamble et al., 2022 [56].
Acute or chronic disease	2	Borgogna et al., 2020 [42], Qulu et al., 2023 [83].
Antibiotic use in the last 30 days	16	Borgogna et al., 2020 [42], Cheng et al., 2024 [38], Li et al., 2024 [59], Liu et al., 2024 [79], Sun et al., 2023 [78], De Magalhães et al., 2022 [58], Choi et al., 2006 [84], Fan et al., 2021 [52], Shi et al., 2024 [75], Feng et al., 2023 [67], Zhaoxi et al., 2021 [81], Kamble et al., 2022 [56], Qulu et al., 2023 [83], Dai et al., 2024 [70], Zhao et al., 2025 [68]
Alcohol or drug dependence	1	Borgogna et al., 2020 [42]
Hormonal therapy	6	Cheng et al., 2024 [38], Liu et al., 2024 [79], Fan et al., 2021 [52], Shi et al., 2024 [75], Dai et al., 2025 [77], Zhao et al., 2025 [68]
Cervical carcinoma	5	Cheng et al., 2024 [62], Li et al., 2024 [59], Li et al., 2024 [63], Choi et al., 2006 [84], Kamble et al., 2022 [56]
Vaginal douching within 48 h	9	Cheng et al., 2024 [38], Li et al., 2024 [59], Sun et al., 2023 [78], Ou et al., 2024 [57], Li et al., 2020 [80], Choi et al., 2006 [84], Fan et al., 2021 [52], Zhaoxi et al., 2021 [81], Dai et al., 2025 [77]
Sexually transmitted infection (STI)	5	Cheng et al., 2024 [38], Ou et al., 2024 [57], Lin et al., 2022 [64], Shi et al., 2024 [75], Kamble et al., 2022 [56]
Sexual activity in the past 72 h	10	Cheng et al., 2024 [38], Li et al., 2024 [59], Ou et al., 2024 [57], Li et al., 2020 [80], Lin et al., 2022 [64], De Magalhães et al., 2022 [58], Shi et al., 2024 [75], Feng et al., 2023 [67], Dai et al., 2025 [77], Zhao et al., 2025 [68]
Severe immune disease	12	Li et al., 2024 [63], Liu et al., 2024 [79], Li et al., 2024 [59], Sun et al., 2023, Lin et al., 2022 [64], Cheng et al., 2024 [38], Fan et al., 2021 [52], Dai et al., 2024 [70], Feng et al., 2023 [67], Zhaoxi et al., 2021 [81], Qulu et al., 2023 [83], Dai et al., 2025 [77]
History of spontaneous abortion	2	Liu et al., 2024 [79], Zhang et al., 2024 [61]
Menopause	1	Liu et al., 2024 [79].
Menstrual period	3	Zhang et al., 2024 [61], Ou et al., 2024 [57], Feng et al., 2023 [67].
Hysterectomy	4	Zhang et al., 2024 [61], Shi et al., 2024 [75], Feng et al., 2023 [67], Zhao et al., 2025 [68].
Vaccinated against HPV	3	Zhang et al., 2024 [61], Li et al., 2024 [63], Feng et al., 2023 [67]
Malignant diseases	2	Li et al., 2024 [63], Shi et al., 2024 [75]
Multiple sexual partners	1	Li et al., 2024 [63]
HIV infection	3	Ou et al., 2024 [57], Dai et al., 2024 [70], Zhao et al., 2025 [68]
Vaginal lubricant or cream	5	Ou et al., 2024 [57], De Magalhães et al., 2022 [58], Dai et al., 2024 [70], Feng et al., 2023 [67], Zhao et al., 2025 [68]
BMI ≥ 30 kg/m^2^	1	Ou et al., 2024 [57]
Endocrine disorders	1	Fan et al., 2021 [52]
Chemotherapy/ radiotherapy	2	Fan et al., 2021 [52], Zhao et al., 2025 [68].

Legend: The background color was added to enhance readability and avoid confusion when interpreting the data.

## References

[1] Bray F., Laversanne M., Sung H., Ferlay J., Siegel R.L., Soerjomataram I., Jemal A. (2024). Global cancer statistics 2022: GLOBOCAN estimates of incidence and mortality worldwide for 36 cancers in 185 countries. CA Cancer J. Clin..

[2] Goldstein A., Gersh M., Skovronsky G., Moss C. (2024). The Future of Cervical Cancer Screening. Int. J. Womens Health.

[3] Nkwabong E., Badjan I.L.B., Sando Z. (2019). Pap smear accuracy for the diagnosis of cervical precancerous lesions. Trop. Dr..

[4] World Health Organization (2021). Regional Implementation Framework for Elimination of Cervical Cancer as a Public Health Problem 2021–2030.

[5] Xing B., Guo J., Sheng Y., Wu G., Zhao Y. (2020). Human Papillomavirus-Negative Cervical Cancer: A Comprehensive Review. Front. Oncol..

[6] Shao N. (2024). Research progress on human papillomavirus-negative cervical cancer: A review. Medicine.

[7] Lee J.E., Chung Y., Rhee S., Kim T.H. (2022). Untold story of human cervical cancers: HPV-negative cervical cancer. BMB Rep..

[8] Pimple S., Mishra G. (2022). Cancer cervix: Epidemiology and disease burden. Cytojournal.

[9] Bruni L., Albero G., Rowley J., Alemany L., Arbyn M., Giuliano A.R., Markowitz L.E., Broutet N., Taylor M. (2023). Global and regional estimates of genital human papillomavirus prevalence among men: A systematic review and meta-analysis. Lancet Glob. Health.

[10] Traynor D., Martin C.M., White C., Reynolds S., D’Arcy T., O’Leary J.J., Lyng F.M. (2021). Raman Spectroscopy of Liquid-Based Cervical Smear Samples as a Triage to Stratify Women Who Are HPV-Positive on Screening. Cancers.

[11] Villa L.L., Richtmann R. (2023). HPV vaccination programs in LMIC: Is it time to optimize schedules and recommendations?. J. Pediatr..

[12] Wang M., Liang H., Yan Y., Bian R., Huang W., Zhang X., Nie J. (2024). Distribution of HPV types among women with HPV-related diseases and exploration of lineages and variants of HPV 52 and 58 among HPV-infected patients in China: A systematic literature review. Hum. Vaccines Immunother..

[13] Abudula A., Rouzi N., Xu L., Yang Y., Hasimu A. (2020). Tissue-based metabolomics reveals potential biomarkers for cervical carcinoma and HPV infection. J. Basic. Med. Sci..

[14] Faktor J., Henek T., Hernychova L., Singh A., Vojtesek B., Polom J., Bhatia R., Polom K., Cuschieri K., Cruickshank M. (2024). Metaproteomic analysis from cervical biopsies and cytologies identifies protein-aceous biomarkers representing both human and microbial species. Talanta.

[15] Platz-Christensen J.J., Sundstrom E., Larsson P.G. (1994). Bacterial vaginosis and cervical intraepithelial neoplasia. Acta Obstet. Gynecol. Scand..

[16] Maarsingh J.D., Łaniewski P., Herbst-Kralovetz M.M. (2022). Immunometabolic and potential tumor-promoting changes in 3D cervical cell models infected with bacterial vaginosis-associated bacteria. Commun. Biol..

[17] Liang Y., Chen M., Qin L., Wan B., Wang H. (2019). A meta-analysis of the relationship between vaginal microecology, human papillomavirus infection and cervical intraepithelial neoplasia. Infect. Agent. Cancer.

[18] Muzny C.A., Cerca N., Elnaggar J.H., Taylor C.M., Sobel J.D., Van Der Pol B. (2023). State of the Art for Diagnosis of Bacterial Vaginosis. J. Clin. Microbiol..

[19] Usyk M., Zolnik C.P., Castle P.E., Porras C., Herrero R., Gradissimo A., Gonzalez P., Safaeian M., Schiffman M., Burk R.D. (2020). Cervicovaginal microbiome and natural history of HPV in a longitudinal study. PLoS Pathog..

[20] Lee J.E., Lee S., Lee H., Song Y.-M., Lee K., Han M.J., Sung J., Ko G. (2013). Association of the Vaginal Microbiota with Human Papillomavirus Infection in a Korean Twin Cohort. PLoS ONE.

[21] Ye J., Qi X. (2023). Vaginal microecology and its role in human papillomavirus infection and human papillomavirus associated cervical lesions. APMIS.

[22] Frąszczak K., Barczyński B., Kondracka A. (2022). Does *Lactobacillus* Exert a Protective Effect on the Development of Cervical and Endometrial Cancer in Women?. Cancers.

[23] Audirac-Chalifour A., Torres-Poveda K., Bahena-Román M., Téllez-Sosa J., Martínez-Barnetche J., Cortina-Ceballos B., López-Estrada G., Delgado-Romero K., I Burguete-García A., Cantú D. (2016). Cervical Microbiome and Cytokine Profile at Various Stages of Cervical Cancer: A Pilot Study. PLoS ONE.

[24] Zheng J.-J., Song J.-H., Yu C.-X., Wang F., Wang P.-C., Meng J.-W. (2019). Difference in vaginal microecology, local immunity and HPV infection among childbearing-age women with different degrees of cervical lesions in Inner Mongolia. BMC Women’s Health.

[25] Bokulich N.A., Łaniewski P., Adamov A., Chase D.M., Caporaso J.G., Herbst-Kralovetz M.M. (2022). Multi-omics data integration reveals metabolome as the top predictor of the cervicovaginal microenvironment. PLoS Comput. Biol..

[26] Mitra A., MacIntyre D.A., Ntritsos G., Smith A., Tsilidis K.K., Marchesi J.R., Bennett P.R., Moscicki A.-B., Kyrgiou M. (2020). The vaginal microbiota associates with the regression of untreated cervical intraepithelial neoplasia 2 lesions. Nat. Commun..

[27] So K.A., Yang E.J., Kim N.R., Hong S.R., Lee J.-H., Hwang C.-S., Shim S.-H., Lee S.J., Kim T.J. (2020). Changes of vaginal microbiota during cervical carcinogenesis in women with human papillomavirus infection. PLoS ONE.

[28] Fang B., Li Q., Wan Z., OuYang Z., Zhang Q. (2022). Exploring the Association Between Cervical Microbiota and HR-HPV Infection Based on 16S rRNA Gene and Metagenomic Sequencing. Front. Cell. Infect. Microbiol..

[29] Bauer G. (2001). Lactobacilli-mediated control of vaginal cancer through specific reactive oxygen species interaction. Med. Hypotheses.

[30] Borella F., Carosso A.R., Cosma S., Preti M., Collemi G., Cassoni P., Bertero L., Benedetto C. (2021). Gut Microbiota and Gynecological Cancers: A Summary of Pathogenetic Mechanisms and Future Directions. ACS Infect. Dis..

[31] Castanheira C.P., Sallas M.L., Nunes R.A.L., Lorenzi N.P.C., Termini L. (2020). Microbiome and Cervical Cancer. Pathobiology.

[32] Dai W., Du H., Li S., Wu R. (2021). Cervicovaginal Microbiome Factors in Clearance of Human Papillomavirus Infection. Front. Oncol..

[33] Ilhan Z.E., Łaniewski P., Thomas N., Roe D.J., Chase D.M., Herbst-Kralovetz M.M. (2019). Deciphering the complex interplay between microbiota, HPV, inflammation and cancer through cervicovaginal metabolic profiling. EBioMedicine.

[34] Srivastava A., Creek D.J. (2018). Discovery and Validation of Clinical Biomarkers of Cancer: A Review Combining Metabolomics and Proteomics. Proteomics.

[35] Hu J., Wu Y., Quan L., Yang W., Lang J., Tian G., Meng B. (2022). Research of cervical microbiota alterations with human papillomavirus infection status and women age in Sanmenxia area of China. Front. Microbiol..

[36] Chorna N., Romaguera J., Godoy-Vitorino F. (2020). Cervicovaginal Microbiome and Urine Metabolome Paired Analysis Reveals Niche Partitioning of the Microbiota in Patients with Human Papilloma Virus Infections. Metabolites.

[37] Jia Y., Zou K., Zou L. (2023). Research progress of metabolomics in cervical cancer. Eur. J. Med Res..

[38] Cheng X., Luo H., Ma J., Wang Y., Su J. (2024). Correlation between Indicators of Vaginal Microbiota and Human Papillomavirus Infection: A Retrospective Study. Clin. Exp. Obstet. Gynecol..

[39] Liu J., Hu N., Zheng X., Li H., Zhao K., Wang J., Zhang M., Zhang L., Song L., Lyu Y. (2024). Vaginal micro-environment disorder promotes malignant prognosis of low-grade cervical intraepithelial neoplasia: A prospective community cohort study in Shanxi Province, China. Clin. Transl. Oncol..

[40] Hu M., Yang W., Yan R., Chi J., Xia Q., Yang Y., Wang Y., Sun L., Li P. (2024). Co-evolution of vaginal microbiome and cervical cancer. J. Transl. Med..

[41] Yang Q., Wang Y., Wei X., Zhu J., Wang X., Xie X., Lu W. (2020). The Alterations of Vaginal Microbiome in HPV16 Infection as Identified by Shotgun Metagenomic Sequencing. Front. Cell. Infect. Microbiol..

[42] Borgogna J., Shardell M.D., Santori E., Nelson T., Rath J., Glover E., Ravel J., Gravitt P., Yeoman C., Brotman R. (2019). The vaginal metabolome and microbiota of cervical HPV-positive and HPV-negative women: A cross-sectional analysis. BJOG Int. J. Obstet. Gynaecol..

[43] Wu M., Yu H., Gao Y., Li H., Wang C., Li H., Ma X., Dong M., Li B., Bai J. (2023). Leveraging 16S rRNA data to uncover vaginal microbial signatures in women with cervical cancer. Front. Cell. Infect. Microbiol..

[44] Peters M.D.J., Godfrey C., McInerney P., Munn Z., Tricco A.C., Khalil H., Aromataris E., Lockwood C., Porritt K., Pilla B., Jordan Z. (2024). Scoping Reviews. JBI Manual for Evidence Synthesis.

[45] Tricco A.C., Lillie E., Zarin W., O’Brien K.K., Colquhoun H., Levac D., Moher D., Peters M.D.J., Horsley T., Weeks L. (2018). PRISMA Extension for Scoping Reviews (PRISMA-ScR): Checklist and Explanation. Ann. Intern. Med..

[46] Ouzzani M., Hammady H., Fedorowicz Z., Elmagarmid A. (2016). Rayyan—A web and mobile app for systematic reviews. Syst. Rev..

[47] Ferreira T., Rasband W. ImageJ User Guide. USA: National Institutes of Health 2011. https://imagej.net/ij/docs/guide/.

[48] R Core Team (2021). R: A Language and Environment for Statistical Computing.

[49] Wickham H. (2016). ggplot2: Elegant Graphics for Data Analysis.

[50] Page M.J., McKenzie J.E., Bossuyt P.M., Boutron I., Hoffmann T.C., Mulrow C.D., Shamseer L., Tetzlaff J.M., Akl E.A., Brennan S.E. (2021). The PRISMA 2020 statement: An updated guideline for reporting systematic reviews. BMJ.

[51] Pawar K., Aranha C. (2022). Lactobacilli metabolites restore E-cadherin and suppress MMP9 in cervical cancer cells. Curr. Res. Toxicol..

[52] Fan Q., Wu Y., Li M., An F., Yao L., Wang M., Wang X., Yuan J., Jiang K., Li W. (2021). *Lactobacillus* spp. create a protective micro-ecological environment through regulating the core fucosylation of vaginal epithelial cells against cervical cancer. Cell Death Dis..

[53] Motevaseli E., Shirzad M., Akrami S.M., Mousavi A.-S., Mirsalehian A., Modarressi M.H. (2013). Normal and tumour cervical cells respond differently to vaginal lactobacilli, independent of pH and lactate. J. Med. Microbiol..

[54] Nicolò S., Tanturli M., Mattiuz G., Antonelli A., Baccani I., Bonaiuto C., Baldi S., Nannini G., Menicatti M., Bartolucci G. (2021). Vaginal Lactobacilli and Vaginal Dysbiosis-Associated Bacteria Differently Affect Cervical Epithelial and Immune Homeostasis and Anti-Viral Defenses. Int. J. Mol. Sci..

[55] Wang K.-D., Xu D.-J., Wang B.-Y., Yan D.-H., Lv Z., Su J.-R. (2017). Inhibitory Effect of Vaginal *Lactobacillus* Supernatants on Cervical Cancer Cells. Probiotics Antimicrob. Proteins.

[56] Kamble A., Naik S., Talathi M., Jadhav D., Pingale S., Kaul-Ghanekar R. (2022). Cervicovaginal microbiota isolated from healthy women exhibit probiotic properties and antimicrobial activity against pathogens isolated from cervical cancer patients. Arch. Microbiol..

[57] Ou J., Kang Y., Medlegeh, Fu K., Zhang Y., Yang W. (2024). An analysis of the vaginal microbiota and cervicovaginal metabolomics in cervical lesions and cervical carcinoma. Heliyon.

[58] de Magalhães C.C.B., Linhares I.M., Masullo L.F., Eleutério R.M.N., Witkin S.S., Eleutério J. (2021). Comparative measurement of D- and L-lactic acid isomers in vaginal secretions: Association with high-grade cervical squamous intraepithelial lesions. Arch. Gynecol. Obstet..

[59] Li J., Jiang L., Wang C.B., Meng J.B., Wang H.B., Jin H.B. (2024). Investigation of the relationship between the changes in vaginal microecological enzymes and human papillomavirus (HPV) infection. Medicine.

[60] Yang X., Shui Y., Qian Y. (2024). A Crosstalk Analysis of high-risk human papillomavirus, microbiota and vaginal metabolome in cervicovaginal microenvironment. Microb. Pathog..

[61] Zhang Y., Wu X., Li D., Huang R., Deng X., Li M., Du F., Zhao Y., Shen J., Chen Y. (2024). HPV-associated cervicovaginal microbiome and host metabolome characteristics. BMC Microbiol..

[62] Cheng L., Yan C., Yang Y., Hong F., Du J. (2024). Exploring the Clinical Signatures of Cervical Dysplasia Patients and Their Association With Vaginal Microbiota. Cancer Med..

[63] Li J., Jin H., Sun Y., Wang C., Chen H., Gong S., Jiang L. (2024). Reconnoitering correlation between human papillomavirus infection-induced vaginal microecological abnormality and squamous intraepithelial lesion (SIL) progression. BMC Women’s Health.

[64] Lin W., Zhang Q., Chen Y., Dong B., Xue H., Lei H., Lu Y., Wei X., Sun P. (2022). Changes of the vaginal microbiota in HPV infection and cervical intraepithelial neoplasia: A cross-sectional analysis. Sci. Rep..

[65] Xu H., Liu L., Xu F., Liu M., Song Y., Chen J., Zhan H., Zhang Y., Xu D., Chen Y. (2022). Microbiome-metabolome analysis reveals cervical lesion alterations. Acta Biochim. Biophys. Sin..

[66] Shen S., Zhao S., Shan J., Ren Q. (2025). Metabolomic and microbiota profiles in cervicovaginal lavage fluid of women with high-risk human papillomavirus infection. Sci. Rep..

[67] Feng F., Hou Y.-M., Zhang Y., Wang L.-Y., Li P.-P., Guo Y., An R.-F. (2023). Correlation analysis of vaginal microecology and different types of human papillomavirus infection: A study conducted at a hospital in northwest China. Front. Med..

[68] Zhao S., Yang H., Lv A., Zhang S., Hui Y., Qi W., Zhao H., Miao M., Wang Y., Yin Y. (2025). Vaginal Microbiome and Metabolome Profiles Among HPV Positive and HPV Negative Women Based on Stratification of Vaginitis. J. Med. Virol..

[69] Jimenez N.R., Mancilla V., Łaniewski P., Herbst-Kralovetz M.M. (2024). Immunometabolic Contributions of Atopobiaceae Family Members in Human Papillomavirus Infection, Cervical Dysplasia, and Cancer. J. Infect. Dis..

[70] Dai W., Du H., Zhou Q., Li S., Wang Y., Hou J., Guo C., Yang Q., Li C., Xie S. (2024). Metabolic profiles outperform the microbiota in assessing the response of vaginal microenvironments to the changed state of HPV infection. NPJ Biofilms Microbiomes.

[71] Zheng J.-J., Miao J.-R., Wu Q., Yu C.-X., Mu L., Song J.-H. (2020). Correlation between HPV-negative cervical lesions and cervical microenvironment. Taiwan. J. Obstet. Gynecol..

[72] Wang P.C., Song J.H. (2017). The correlation between vaginal microecological changes and HPV outcome in patients with cervical lesions in the Inner Mongolia area of China. Int. J. Clin. Exp. Med..

[73] Zhang H., Jin S., Ji A., Zhang C., Shi S. (2022). Correlation between Vaginal Microecological Status and Prognosis of CIN Patients with High-Risk HPV Infection. BioMed Res. Int..

[74] Tao H., Zeng D., Chen W., Li F., Zhong H., Fu J., Liu H., Ying T., Wang L., Chen J. (2023). Focused ultrasound: A novel therapy for improving vaginal microecology in patients with high-risk HPV infection. Int. J. Hyperth..

[75] Shi Y., Dong X.Y., Yimingjiang M.W.L.D., Ma W.M., Ma Z.P., Pang X.L., Zhang W. (2024). The association between human papillomavirus infection, vaginal microecology, and cervical intraepithelial neoplasia in women from Xinjiang, China. J. Obstet. Gynaecol. Res..

[76] Dasari S., Rajendra W., Valluru L. (2014). Evaluation of Microbial Enzymes in Normal and Abnormal Cervicovaginal Fluids of Cervical Dysplasia: A Case Control Study. BioMed Res. Int..

[77] Dai W., Liu Y., Jiang X., Xu R., Guo C., Hou J., Wu D., Li C., Du H., Wu R. (2025). The inferred modulation of correlated vaginal microbiota and metabolome by cervical differentially expressed genes across distinct CIN grades. BMC Microbiol..

[78] Ma C., Sun L., Li L., Xu W. (2023). The Immunomodulation Role of Vaginal Microenvironment On Human Papillomavirus Infection. Galen Med J..

[79] Liu H.-M., Zhang F., Cai H.-Y., Lv Y.-M., Pi M.-Y. (2024). Cross-Sectional Study on the Correlation Between Vaginal Microecology and High-Risk Human Papillomavirus Infection: Establishment of a Clinical Prediction Model. Int. J. Women’s Health.

[80] Li L., Ding L., Gao T., Lyu Y., Wang M., Song L., Li X., Gao W., Han Y., Jia H. (2020). Association between Vaginal Micro-environment Disorder and Cervical Intraepithelial Neoplasia in a Community Based Population in China. J. Cancer.

[81] Lu Z., Sun B., Zhang D. (2021). Human papillomavirus genotyping and vaginal microbial metabolites in 276 patients with atypical cervical squamous cells and the clinical effect of nano-silver after loop electrosurgical excision procedure. Mater. Express.

[82] Venancio P.A., Consolaro M.E.L., Derchain S.F., Boccardo E., Villa L.L., Maria-Engler S.S., Campa A., Discacciati M.G. (2019). Indoleamine 2,3-dioxygenase and tryptophan 2,3-dioxygenase expression in HPV infection, SILs, and cervical cancer. Cancer Cytopathol..

[83] Qulu W., Mtshali A., Osman F., Ndlela N., Ntuli L., Mzobe G., Naicker N., Garrett N., Rompalo A., Mindel A. (2023). High-risk human papillomavirus prevalence among South African women diagnosed with other STIs and BV. PLoS ONE.

[84] Choi H.S., Kim K.M., Kim C.H., Kim S.M., Oh J.S. (2006). Hydrogen Peroxide Producing Lactobacilli in Women with Cervical Neoplasia. Cancer Res. Treat..

[85] Xu H., Zhang S., Zhang B., Jiang N., Xu Y., Chen X., Han L. (2024). Vaginal colonization of Lactobacilli: Mechanism and function. Microb. Pathog..

[86] Kumar K.P., Reddi V. (2023). Significance of Spearman’s rank correlation coefficient. Int. J. Multidiscip. Res..

[87] Schober P., Boer C., Schwarte L.A. (2018). Correlation Coefficients: Appropriate Use and Interpretation. Anesth. Analg..

[88] Wang H., Ma Y., Li R., Chen X., Wan L., Zhao W. (2019). Associations of Cervicovaginal Lactobacilli With High-Risk Human Papillomavirus Infection, Cervical Intraepithelial Neoplasia, and Cancer: A Systematic Review and Meta-Analysis. J. Infect. Dis..

[89] Alizhan D., Ukybassova T., Bapayeva G., Aimagambetova G., Kongrtay K., Kamzayeva N., Terzic M. (2025). Cervicovaginal Microbiome: Physiology, Age-Related Changes, and Protective Role Against Human Papillomavirus Infection. J. Clin. Med..

[90] Alimena S., Davis J., Fichorova R.N., Feldman S. (2022). The vaginal microbiome: A complex milieu affecting risk of human papillomavirus persistence and cervical cancer. Curr. Probl. Cancer.

[91] Kudela E., Liskova A., Samec M., Koklesova L., Holubekova V., Rokos T., Kozubik E., Pribulova T., Zhai K., Busselberg D. (2021). The interplay between the vaginal microbiome and innate immunity in the focus of predictive, preventive, and personalized medical approach to combat HPV-induced cervical cancer. EPMA J..

[92] Mitra A., MacIntyre D.A., Marchesi J.R., Lee Y.S., Bennett P.R., Kyrgiou M. (2016). The vaginal microbiota, human papillomavirus infection and cervical intraepithelial neoplasia: What do we know and where are we going next?. Microbiome.

[93] Di Paola M., Sani C., Clemente A.M., Iossa A., Perissi E., Castronovo G., Tanturli M., Rivero D., Cozzolino F., Cavalieri D. (2017). Characterization of cervico-vaginal microbiota in women developing persistent high-risk Human Papillomavirus infection. Sci. Rep..

[94] A Vodstrcil L., Hocking J.S., Law M., Walker S., Tabrizi S.N., Fairley C.K., Bradshaw C.S. (2013). Hormonal Contraception Is Associated with a Reduced Risk of Bacterial Vaginosis: A Systematic Review and Meta-Analysis. PLoS ONE.

[95] Bradley F., Birse K., Hasselrot K., Noël-Romas L., Introini A., Wefer H., Seifert M., Engstrand L., Tjernlund A., Broliden K. (2018). The vaginal microbiome amplifies sex hormone-associated cyclic changes in cervicovaginal inflammation and epithelial barrier disruption. Am. J. Reprod. Immunol..

[96] Pendharkar S., Skafte-Holm A., Simsek G., Haahr T. (2023). Lactobacilli and Their Probiotic Effects in the Vagina of Reproductive Age Women. Microorganisms.

[97] France M., Alizadeh M., Brown S., Ma B., Ravel J. (2022). Towards a deeper understanding of the vaginal microbiota. Nat. Microbiol..

[98] Saraf V.S., Sheikh S.A., Ahmad A., Gillevet P.M., Bokhari H., Javed S. (2021). Vaginal microbiome: Normalcy vs. dysbiosis. Arch. Microbiol..

[99] Krüger H., Bauer G. (2017). Lactobacilli enhance reactive oxygen species-dependent apoptosis-inducing signaling. Redox Biol..

[100] Vaneechoutte M. (2017). The human vaginal microbial community. Res. Microbiol..

[101] Ebrahimi S., Soltani A., Hashemy S.I. (2018). Oxidative stress in cervical cancer pathogenesis and resistance to therapy. J. Cell. Biochem..

[102] Despot A., Fureš R., Despot A.-M., Mikuš M., Zlopaša G., D’aMato A., Chiantera V., Serra P., Etrusco A., Laganà A.S. (2023). Reactive oxygen species within the vaginal space: An additional promoter of cervical intraepithelial neoplasia and uterine cervical cancer development?. Open Med..

[103] Tachedjian G., O’hAnlon D.E., Ravel J. (2018). The implausible “in vivo” role of hydrogen peroxide as an antimicrobial factor produced by vaginal microbiota. Microbiome.

[104] Dong M., Dong Y., Bai J., Li H., Ma X., Li B., Wang C., Li H., Qi W., Wang Y. (2023). Interactions between microbiota and cervical epithelial, immune, and mucus barrier. Front. Cell. Infect. Microbiol..

[105] Sun S., Li H., Chen J., Qian Q. (2017). Lactic Acid: No Longer an Inert and End-Product of Glycolysis. Physiology.

[106] Yang X., Da M., Zhang W., Qi Q., Zhang C., Han S. (2018). Role of *Lactobacillus* in cervical cancer. Cancer Manag. Res..

[107] Hearps A., Tyssen D., Srbinovski D., Bayigga L., Diaz D.J.D., Aldunate M., Cone R., Gugasyan R., Anderson D., Tachedjian G. (2017). Vaginal lactic acid elicits an anti-inflammatory response from human cervicovaginal epithelial cells and inhibits production of pro-inflammatory mediators associated with HIV acquisition. Mucosal Immunol..

[108] Torcia M.G. (2019). Interplay among Vaginal Microbiome, Immune Response and Sexually Transmitted Viral Infections. Int. J. Mol. Sci..

[109] Läsche M., Urban H., Gallwas J., Gründker C. (2021). HPV and Other Microbiota; Who’s Good and Who’s Bad: Effects of the Microbial Environment on the Development of Cervical Cancer—A Non-Systematic Review. Cells.

[110] Vitali B., Cruciani F., Picone G., Parolin C., Donders G., Laghi L. (2015). Vaginal microbiome and metabolome highlight specific signatures of bacterial vaginosis. Eur. J. Clin. Microbiol. Infect. Dis..

[111] Challa A., Maras J.S., Nagpal S., Tripathi G., Taneja B., Kachhawa G., Sood S., Dhawan B., Acharya P., Upadhyay A.D. (2024). Multi-omics analysis identifies potential microbial and metabolite diagnostic biomarkers of bacterial vaginosis. J. Eur. Acad. Dermatol. Venereol..

[112] Yang K., Xia B., Wang W., Cheng J., Yin M., Xie H., Li J., Ma L., Yang C., Li A. (2017). A Comprehensive Analysis of Metabolomics and Transcriptomics in Cervical Cancer. Sci. Rep..

[113] Weaver C., Nam A., Settle C., Overton M., Giddens M., Richardson K.P., Piver R., Mysona D.P., Rungruang B., Ghamande S. (2024). Serum Proteomic Signatures in Cervical Cancer: Current Status and Future Directions. Cancers.

[114] Huang X., Li X., Li S., Wu J., Duan Z., Luo M., Jia Y. (2025). Vaginal metabolic profiling reveals biomarkers characteristics of high-risk HPV infection and cervical lesions. J. Obstet. Gynaecol. Res..

[115] Jeong J., Yun K., Mun S., Chung W.-H., Choi S.-Y., Nam Y.-D., Lim M.Y., Hong C.P., Park C., Ahn Y.J. (2021). The effect of taxonomic classification by full-length 16S rRNA sequencing with a synthetic long-read technology. Sci. Rep..

[116] Morsli M., Gimenez E., Magnan C., Salipante F., Huberlant S., Letouzey V., Lavigne J.-P. (2024). The association between lifestyle factors and the composition of the vaginal microbiota: A review. Eur. J. Clin. Microbiol. Infect. Dis..

[117] Amabebe E., Tatiparthy M., Kammala A.K., Richardson L.S., Taylor B.D., Sharma S., Menon R. (2025). Vaginal pharmacomicrobiomics modulates risk of persistent and recurrent bacterial vaginosis. NPJ Biofilms Microbiomes.

[118] Kaur H., Merchant M., Haque M.M., Mande S.S. (2020). Crosstalk Between Female Gonadal Hormones and Vaginal Microbiota Across Various Phases of Women’s Gynecological Lifecycle. Front. Microbiol..

[119] Lebeer S., Ahannach S., Gehrmann T., Wittouck S., Eilers T., Oerlemans E., Condori S., Dillen J., Spacova I., Donck L.V. (2023). A citizen-science-enabled catalogue of the vaginal microbiome and associated factors. Nat. Microbiol..

[120] Song S.D., Acharya K.D., Zhu J.E., Deveney C.M., Walther-Antonio M.R.S., Tetel M.J., Chia N. (2020). Daily Vaginal Microbiota Fluctuations Associated with Natural Hormonal Cycle, Contraceptives, Diet, and Exercise. mSphere.

